# Going Beyond the Limits of Classical Atomistic Modeling of Plasmonic Nanostructures

**DOI:** 10.1021/acs.jpcc.1c04716

**Published:** 2021-10-26

**Authors:** Piero Lafiosca, Tommaso Giovannini, Michele Benzi, Chiara Cappelli

**Affiliations:** Scuola Normale Superiore, Piazza dei Cavalieri 7, 56126 Pisa, Italy

## Abstract

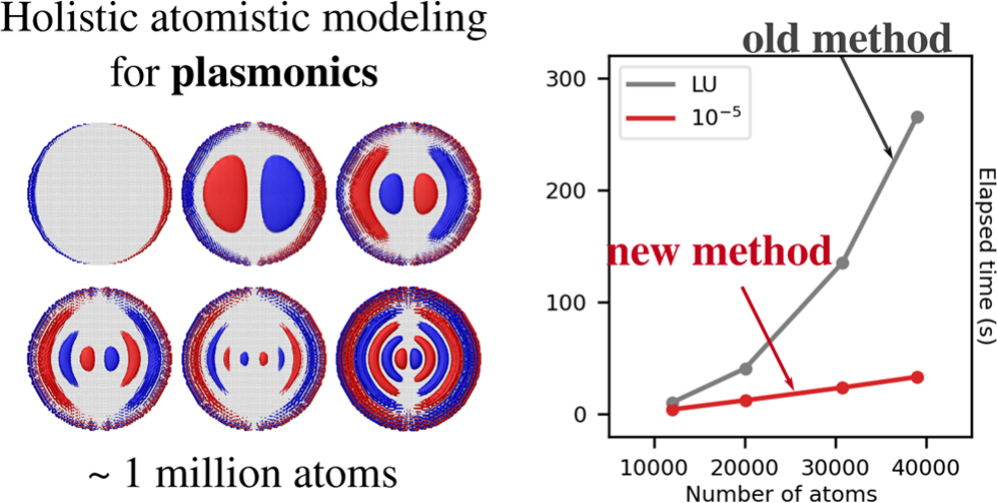

Theoretical modeling of plasmonic phenomena is of fundamental importance for rationalizing experimental measurements. Despite the great success of classical continuum modeling, recent technological advances allowing for the fabrication of structures defined at the atomic level require to be modeled through atomistic approaches. From a computational point of view, the latter approaches are generally associated with high computational costs, which have substantially hampered their extensive use. In this work, we report on a computationally fast formulation of a classical, fully atomistic approach, able to accurately describe both metal nanoparticles and graphene-like nanostructures composed of roughly 1 million atoms and characterized by structural defects.

## 1 Introduction

Nanoplasmonics is an emerging field that has significantly developed in the last decade.^1,2^ Free-electron nanomaterials, such as metal nanoaggregates or graphene, are characterized by the rise of surface plasmons, i.e., coherent oscillations of the conduction electrons that are induced by external radiation.^3^ One of the peculiarities of such materials is that their plasmon resonance frequency (PRF) can be tuned as a function of nanostructure’s shape, size, and supramolecular structure.^4−7^ In the particular case of graphene, PRF can also be tuned through electrical gating and chemical doping, which modify the Fermi energy level; such a peculiarity is exploited in many diverse applications, as well as in the technological field.^8^

As a matter of fact, small-size graphene-based nanostructures (with a typical dimension lower than 5 nm) do not experimentally exhibit the same plasmonic properties as large structures;^9^ therefore, only the latter are actually exploited in real applications. Moreover, the typical size of metal nanoparticles exploited in many applications (as, for instance, surface-enhanced Raman scattering—SERS) is of several tens of nanometers.^10^ The necessity of treating large structures strongly limits the applicability of full quantum mechanical (QM) descriptions, which are totally impracticable for real-size systems. For this reason, plasmonic structures are generally simulated by resorting to classical approaches,^11−20^ and in particular through continuum models that describe plasmonic materials as a function of their frequency-dependent complex permittivity function.^21−27^

Continuum models have played a fundamental role in the study of nanostructured plasmonic systems, thanks to the excellent compromise between accuracy and low computational cost.^11−17,28,29^ However, recent technological advances in the manufacturing of nanostructured materials have exposed their limitations. In fact, it is nowadays possible to achieve fine structural control, down to the atomic limit, and in such cases, the strong approximation on which continuum approaches are based is not justified.^10^ Therefore, alternative approaches are required, and a promising solution is to resort to fully atomistic, yet classical, models, which combine a fine structural resolution of the nanostructure at the atomic level, with a reasonable computational cost.^11−20^

In addition, atomistic modeling permits the treatment of structural defects, doping, and in general of local structural anisotropies, which cannot be described by a continuum approach and which can tune the plasmonic properties of the whole structure. Geometrical strain and subnanometer junctions, for instance, occurring in tip-enhanced Raman scattering (TERS), are two examples of geometrical anisotropies that need atomistic approaches to be reliably modeled. However, the greater level of detail obtained by atomistic models is usually accompanied by higher computational costs, which have hampered so far their massive use in the modeling of plasmonic phenomena, that are mainly due to the fact that computationally tractable structures are much smaller in size than those experimentally studied.

As stated above, to enhance the applicability of atomistic modeling toward realistic nanoplasmonic materials, it is crucial to increase the size of treatable structures up to those that are routinely experimentally investigated, which are usually composed of few million atoms. To address such a problem, in this paper, we propose for the first time a holistic fully atomistic, yet classical, ω-fluctuating charges (ωFQ) approach,^28−30^ which is remarkably able to treat at the same time and with the same level of accuracy three-dimensional (3D) plasmonic systems (metal nanostructures) and two-dimensional (2D) materials.

The novel approach is formulated analogously with the most widely used approaches based on implicit descriptions (e.g., boundary element method (BEM)^25,31^), thus proposing for the first time a fully atomistic treatment of the same physical features (including the potential extension to interband transitions). In addition, we demonstrate for the first time the uniqueness and existence of the solution to the ωFQ problem, which is a fundamental prerequisite for any consistent physical model. In fact, it allows to exploit state-of-the-art numerical methods to solve the ωFQ problem, with a significant reduction in the computational timings and also in memory requirements. Last, but not least, the present reformulation and the consequent implementation allow the first fully atomistic, yet classical, calculations of the plasmonic properties of structures composed of ∼1 million atoms, also bearing structural defects, which significantly affect their plasmonic response.

In ωFQ, each atom of the nanosubstrate is endowed with a charge, the value of which depends on the external frequency ω and is determined by solving a complex-valued linear system.^28−30^ The charge exchange between atoms is governed by the Drude mechanism and is limited to nearest neighbors by applying a Fermi-like damping function, which recovers the typical behavior of quantum tunneling, that is crucial to reproduce the charge flow in subnanometer junctions. In previous works, we demonstrated that ωFQ is able to almost perfectly reproduce reference ab initio, continuum, and experimental data for metal nanoparticles^28,30^ and graphene-based nanostructures.^29^

The manuscript is organized as follows. In the next section, we briefly recap the fundamentals of ωFQ and present a novel approach that treats the plasmonic properties of metal nanoparticles and graphene-based nanostructures within a unified framework. We then apply the new algorithms to selected 2D and 3D nanostructures of sizes up to hundreds of nanometers and constituted by roughly one million atoms. A brief summary and an overview of future developments end the manuscript.

## 2 Methods

### 2.1 ωFQ: A Unified Approach for 2D and 3D Plasmonic Nanostructures

ωFQ is a fully atomistic, classical model that describes the response of metal nanoparticles or graphene-based nanostructures to the external electric field **E**.^28^ Each atom of the system is endowed with a charge, and charge exchange between different atoms is governed by the Drude mechanism of conduction^32^ and modulated by quantum tunneling.^28^ The key equation for solving the charges **q** reads
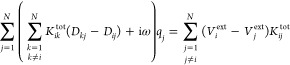
1where *V*_*i*_^ext^ is the electric potential acting on the *i*th charge associated with the external electric field oscillating at frequency ω, *D*_*ij*_ is the charge−charge interaction kernel, and *K*_*ij*_^tot^ is a matrix accounting for both Drude and tunneling mechanisms.

More in detail, the linear system in eq 1 describes the response of a set of *N* complex-valued charges *q*_*j*_ under the effect of an external monochromatic uniform electric field of frequency ω polarized along the **k̂** direction, with **k̂** = **x̂**, **ŷ**, **ẑ**. The associated potential *V*^ext^ on each atom, entering the right-hand side of eq 1, is defined as
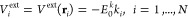
2where *E*_0_^*k*^ is the intensity of the electric field along the *k* direction, **r**_*i*_ is the position of the *i*th charge, and *k*_*i*_ is the component of **r**_*i*_ along the **k̂**-axis, i.e., *k*_*i*_ = **r**_*i*_ ·**k̂**. The matrix **D** on the left-hand side of eq 1 describes the electrostatic interaction between the charges, and it is defined in the standard formulation of the FQ force field exploited for treating molecular systems.^33,34^ To avoid the so-called “polarization catastrophe”,^11^ instead of point charge, we use spherical Gaussian charge distributions of widths *d*_*i*_ and *d*_*j*_ to describe ωFQ charges. The **D** elements then read^28,35−37^
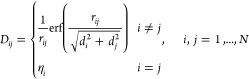
3where *r*_*ij*_ = |**r**_*i*_ – **r**_*j*_| is the distance between charges *i*th and *j*th, erf is the error function, and η_*i*_ is the atomic chemical hardness of the *i*th atom.^35,38^ Gaussian widths *d*_*i*_ and *d*_*j*_ are chosen for each atom by imposing that the limit for **r**_*i*_ → **r**_*j*_ corresponds to the diagonal element of the matrix, i.e.,
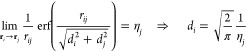
4The **D** matrix can be formally seen as an overlap matrix defined in the scalar product weighted by the  function. Therefore, it is symmetric positive definite (SPD).^35^ The **K**^tot^ matrix in eq 1 reads
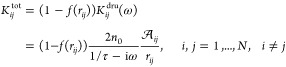
5where *n*_0_ is the electron density, τ is a frictionlike constant due to scattering events, and  is an effective area dividing atoms *i* and *j. f* is a Fermi-like damping function, defined as
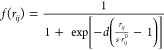
6in which r_*ij*_^0^ is the equilibrium distance between atoms *i* and *j*, while *d* and *s* are parameters ruling the shape of the damping function. **K**^tot^ is a frequency-dependent symmetric complex-valued matrix, and it can be interpreted as a “dynamic” response matrix, whereas the **D** matrix describes the “static” response. It is worth noticing the expression of **K**^tot^ can be associated with two alternative response regimes. When *f*(*r*_*ij*_) goes to zero, the purely Drude conductive regime is recovered; as *r*_*ij*_ increases, the electron transfer decreases exponentially, thus leading to the typical tunneling mechanism.^28^ The diagonal elements of **K**^tot^ do not enter eq 1, but the notation can be simplified by imposing *K*_*ii*_^tot^ = 0 for all *i* = 1,...,*N* and extending the summations over *k* and *j* in eq 1 to all *N* atoms of the system.

The electron density *n*_0_, that appears in eq 5, is a specific property of the chemical composition of the plasmonic substrate and of the shape of the system. In a general 3D system, *n*_0_ can be expressed as , where σ_0_ is the static conductance of the material, while m* is its effective electron mass, which can be approximated to 1 for metal nanoparticles.^39^ However, in the case of graphene-based materials, such as graphene sheets or carbon nanotubes, the effective electron mass needs to be taken into account.^29^ In graphene-based materials, m* can be expressed as
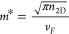
7where *n*_2D_ is the 2D electron density of the system and *v*_*F*_ is the Fermi velocity.^8^ The latter is related to the Fermi energy ε_F_ through expression , where *m*_0_ is the electron rest mass.^8^ The 2D electron density *n*_2D_ can be calculated from *n*_0_ as *n*_2D_ = *n*_0_·*a*_0_, with *a*_0_ being the Bohr radius.^29^ Then, the 2D electron density can be calculated as the ratio of the number of atoms *N* and the surface of the system *S*, i.e.,
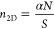
8where α is a parameter (<1) that selects the fraction of π electrons that are involved in the studied plasmonic excitation.^29^ We note that such a parameter is uniquely determined by the value of ε_F_, which can be directly recovered from experimental conditions.^40^

Let us analyze the mathematical properties of the ωFQ linear system defined in eq 1. We first notice that in eq 5 the complex frequency-dependent ratio describing the Drude model can be gathered
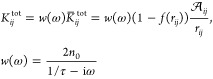
9where **K̅**^tot^ is a symmetric real-valued matrix. The frequency-dependent complex factor *w*(ω) is always nonzero, so we can take it out from eq 1
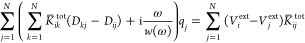
10At this point, we can introduce the following notation
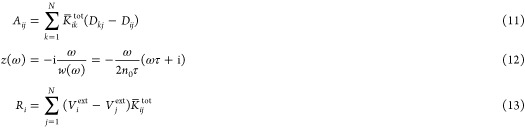
and eq 10 can be expressed in vector notation as
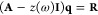
14where **I** is the *N*-dimensional identity matrix. It can be noted that eq 14 is fully equivalent to eq 1, but this time **A** is a real-valued frequency-independent nonsymmetric matrix, of which the diagonal elements are shifted by a complex quantity. Moreover, the **A** matrix defined in eq 11 can be rewritten as
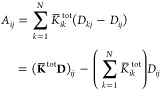
15By introducing a diagonal matrix *P*_*il*_ = (∑_*k*=1_^*N*^*K̅*_*ik*_^tot^) δ_*il*_, where δ_*il*_ is the Kronecker delta, we can write
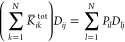
16and plugging the definition into eq 15 we obtain
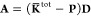
17Therefore, the **A** matrix can be formulated as the product of two real-valued symmetric matrices since **K̅**^tot^ and **D** are symmetric and **P** is diagonal. However, **A** is a nonsymmetric matrix because **D** and **K̅**^tot^ – **P**, in general, do not commute. Nevertheless, the following equality holds
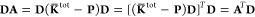
18where ^*T*^ indicates the transposition operator. Recalling that **D** is an SPD matrix,^35^ we can define the **D**-inner product as
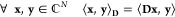
19where ⟨·,·⟩ is the standard Euclidean inner product. From eqs 18 and 19, it can be demonstrated that **A** is self-adjoint with respect to the **D**-inner product, i.e.,
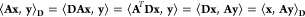
20Equation 20 allows us to conclude that even if **A** is a nonsymmetric matrix, it is self-adjoint with respect to the inner product induced by the SPD matrix **D**; therefore, **A** is diagonalizable with real eigenvalues.

As a final remark, the alternative expression of the **A** matrix in eq 17 allows us to derive another property of the matrix itself. In fact, **K̅**^tot^ – **P** is such that each row (or column) sums up to zero; therefore, it is a singular matrix. By this, the matrix **A** is also singular.

Nevertheless, the existence and uniqueness of the linear system solution in eq 14 are guaranteed through the diagonal shift of the coefficient matrix with the complex scalar *z*(ω) defined in eq 12, which is nonzero when ω ≠ 0. However, numerical instabilities in the solution of the linear system can arise when ω approaches zero because **A** is close to singular.

### 2.2 Preconditioning of the ωFQ Linear System

To model the optical spectra of plasmonic substrates, eq 1, or equivalently eq 14, needs to be solved for a certain number of frequencies ω. This can be effectively achieved by resorting to efficient methods to solve the dense nonsymmetric complex linear system. This point is crucial, especially when the dimensionality of the system increases. Direct techniques of solution (e.g., based on factorization of the coefficient matrix) are to be avoided because they are inefficient both in terms of storage demand and computational cost, which scales as *N*^3^. Thus, matrix-free iterative techniques, which in our implementation scale as *N*^2^, are a promising alternative. In particular, such approaches can be implemented without necessarily storing in memory the whole matrix **A** – *z*(ω) **I** but in terms of matrix–vector products that can efficiently be computed in a parallel environment.

The convergence of iterative techniques can be accelerated (i.e., the number of iterations to reach the solution can be reduced) by exploiting a preconditioner  that, for our specific problem, is defined such that . The linear system in eq 14 can be transformed by applying the preconditioner to the left and/or the right of the coefficient matrix. Thus, we can define a *left-preconditioned* linear system as
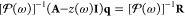
21or a *right-preconditioned* linear system as
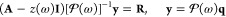
22As it can be easily noticed, the preconditioned linear systems are formally equivalent to the original linear system, but if the preconditioner  is chosen wisely, the convergence rate of the iterative algorithm can be strongly improved. In this work, three different preconditioning strategies have been tested based on the shape of the matrix **A** – *z*(ω) **I** and on the physical properties of the plasmonic substrate. The results are amply discussed in Section S1 in the Supporting Information (SI). In brief, we have exploited (i) a band preconditioner, in which a certain number of supra- and subdiagonals of the **A** matrix are retained, (ii) a symmetric Gauss–Seidel preconditioner,^41−43^ and (iii) a “nearest-neighbors” preconditioner, in which only the matrix elements of **A** – *z*(ω) **I** associated with atoms close in the space are retained. The most promising approach is the band preconditioner (see the SI). However, it is strongly dependent on atom indexing in the construction of the **A** matrix, and, most importantly, it is not of general applicability (see Section S1.1.1 in the SI) because it shows different performance when applied to 2D or 3D systems. We also notice that even when the number of iterations is reduced, the additional computational cost required at each step of the iterative procedure makes the solution of the linear system much more expensive, in terms of both storage and timing, with respect to the non-preconditioned system.

For the aforementioned reasons, in the following discussion, the results obtained by exploiting the non-preconditioned linear system are reported.

### 2.3 Comparison with Continuum Approaches

The atomistic nature of ωFQ emerges from all of the variables that enter eq 1: charge positions, chemical hardnesses in eq 3, effective areas in eq 5, and equilibrium distances in eq 6, as well as electronic features of the material that enter the Drude model. Such an approach allows us to describe the (macroscopic) plasmonic response of the system in terms of (microscopic) atomistic quantities, regardless of the shape of the system. Therefore, complex effects associated with surface roughness and edge effects are automatically considered.

As stated in Section 1, the plasmonic response of complex systems can be described by resorting to continuum approaches, such as the boundary element method (BEM).^25,31^ There, the material is treated as a continuum and its electronic properties are synthesized by its frequency-dependent dielectric permittivity function ε(ω). The plasmonic response arises as a surface charge density σ(**r**), which is computed by solving Maxwell equations, *via* a reformulation as a boundary integral equation on the material surface. From the computational point of view, the latter is discretized in *N* surface elements centered at positions **r**_*i*_, with *i* = 1,...,*N*. At the same time, the surface charge density σ(**r**) is discretized in terms of *N* electric charges. The equation for solving the charges in BEM reads^25^
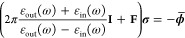
23where ε_out_ and ε_in_ are the frequency-dependent complex-valued dielectric permittivity functions of the outer (usually vacuo) and inner (the material) regions, respectively, σ_*i*_ = σ(**r**_*i*_) is the electric charge evaluated on the surface element at position **r**_*i*_, while  is the surface derivative of the external potential ϕ at position **r**_*i*_. Moreover, **I** is the *N*-dimensional identity matrix and *F*_*ij*_ is the normal derivative of the Green function, i.e.,
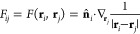
24where **n̂**_*i*_ is the normal vector to the surface at point **r**_*i*_.

ωFQ and BEM are genuinely different in terms of performance and versatility: surface roughness can easily be taken into account through an atomistic approach, while the continuum model needs specific treatments such as perturbative expansions.^44^ Nevertheless, from the purely algebraic point of view, there are some similarities. First, eqs 14 and 23 have the same structure. In both cases, charges are obtained by solving a dense system of linear equations, in which the left-hand side is written in terms of a nonsymmetric frequency-independent real-valued matrix [**A** for ωFQ (see eq 11) and **F** for BEM (see eq 24)] with a uniform complex-valued diagonal shift, which describes the electronic properties of the material at a specific frequency [*z*(ω) defined in eq 12 for ωFQ and the permittivity in eq 23 for BEM]. Moreover, it has been shown that the **F** matrix in eq 24 is singular and diagonalizable with real eigenvalues,^25,45−47^ similar to the **A** matrix in eq 11 (see Section 2.1 for the proof). Therefore, the same computational techniques to solve the linear system, which in this paper are described for the ωFQ approach, can also be exploited for BEM.

## 3 Results and Discussion

To solve the complex-valued ωFQ linear system (see eq 14), the generalized minimal residual (GMRES) and quasi-minimum residual (QMR) algorithms (see the Appendix 1) have been exploited and implemented in a standalone FORTRAN95 code, named nanoFQ, in a parallel environment through the OPENMP application programming interface (API).^48^ To apply complex GMRES, nanoFQ has been interfaced with a public domain software developed by Frayssé and co-workers.^49^ The QMR-from-BiConjugate Gradients (BCG) algorithm without look-ahead for **J**-symmetric matrices^50,51^ has been implemented from scratch. All calculations have been performed on a Xeon Gold 5120 (56 cores, 2.2 GHz) cluster node equipped with 128 GB RAM, if not stated otherwise.

The performance of GMRES and QMR algorithms has been computationally compared by calculating the number of iterations (NI) required to converge the solution of the linear system to a predefined threshold. The 2-norm of the residual vector has been used as a convergence criterion
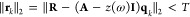
25where **q**_*k*_ is the vector generated at the *k*th iterative step and *T* is a user-defined threshold.

**Figure 1:**
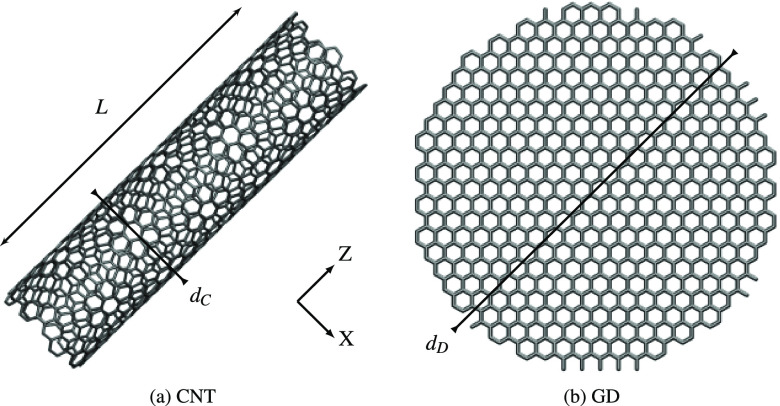
Graphical depiction of CNT (a) and GD (b) molecular structures. The CNT length and diameter (*L*, *d*_C_) and the GD diameter (*d*_D_) are also highlighted.

The ωFQ approach has been applied to the prediction of the optical properties of selected chiral carbon nanotubes (CNTs) and graphene disks (GDs) (see Figure 1a and b for their molecular structures). For both systems, different geometries have been generated by modifying the length *L* and/or the diameter *d*_C_ for CNTs and the diameter *d*_D_ for GDs. The total number of atoms in the studied structures varies from 8208 to 49 248. It is worth remarking that due to the cutting procedure adopted to construct the GDs, dangling bonds possibly occurring on the edges of the disks may be retained (see Figure 1b). However, they do not affect the optical properties of large systems (see Figure S9 in the SI). Thus, they can be retained without affecting computed properties and the convergence rate of the two algorithms.

In the following, we study the dependence of NI on

- electronic parameters, such as the relaxation time τ and the Fermi energy ε_F_ that enter eqs 5 and 7, respectively;
- external field frequency ω, which enters eq 14 through the *z*(ω) coefficient defined in eq 12;
- geometry of the systems, in particular, the GD diameter and the CNT length/diameter (see Figure 1a,b); and
- iterative algorithm, i.e., QMR, GMRES, or restarted GMRES(*k*) (see the Appendix 1).

Since we are dealing with iterative procedures, also the choice of the initial vector **q**_0_ (see eq 25) can strongly affect the NI. Therefore, to compare the different algorithms reliably and in a reproducible way, we choose **q**_0_ = 0 in all cases.

### 3.1 How Electronic Parameters Affect Plasmonic Response and Computational Timings?

We first analyze how the number of iterations to achieve convergence depends on the choice of electronic parameters that enter the definition of ωFQ model, i.e., the Fermi energy ε_F_ (see eq 7) and the relaxation time τ (see eq 5). The plasmonic response of GD varies as a function of both τ and ε_F_.^9,29,52,53^ To analyze such a response, we consider the longitudinal absorption cross section σ_*k*_, which can be calculated as
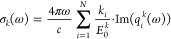
26where *c* is the speed of light, *k*_*i*_ is the position of the *i*th charge along the **k̂**-axis, and *E*_0_^*k*^ is the *k*th component of the intensity of the external electric field. Im(*q*_*i*_^*k*^(ω)) is the imaginary part of the *i*th charge induced by an external electric field polarized along the **k̂**-axis with frequency ω.

It has been shown by some of the present authors^29^ that PRFs, i.e., frequencies corresponding to σ_*k*_ maxima, are independent of τ. In fact, the latter only affects the excitation peak broadening and amplitude, which scale with τ and , respectively.

**Figure 2:**
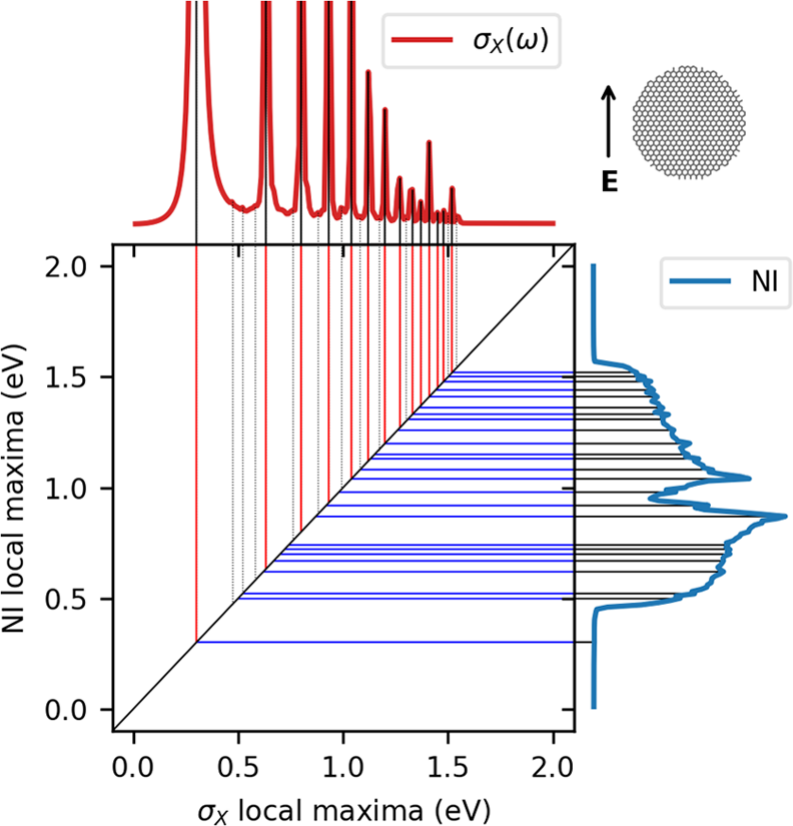
Correlation map between GD20 σ_*X*_(ω) (top plot, red line) and NI (right plot, blue line) local maxima.

The dependence of NI on τ and ε_F_ has been studied for a GD with *d*_D_ = 20 nm (GD20), which is constituted by 11 970 carbon atoms. The full (i.e., nonrestarted) GMRES NI has been calculated on 200 frequencies in the range between 0.01 and 2.0 eV with a constant step of 0.01 eV. The convergence threshold *T* has been fixed to 10^–6^ a.u. (see eq 25). ωFQ parameters have been set to those exploited in refs (29) and (53). The ωFQ linear system has been solved with the GMRES algorithm using ε_F_ = 1.51 eV and τ = 17 000 a.u. We remark that such τ value is typical of graphene sheets.^53^ The computed σ_*X*_(ω) and NI are reported in Figure 2, where the absorption cross section has been scaled to make all peaks visible (see Figure S10 in the SI).

By varying the external field frequency, σ_*X*_ shows a set of local maxima of different amplitudes. Since all calculations have been performed with a constant step of 0.01 eV, each PRF has an intrinsic error of 0.01 eV. Similar errors can also affect the relative intensity of the local maxima: the absorption peak is extremely sharp when τ is large; thus, a small variation in frequency induces a large variation in intensity.

**Figure 3:**
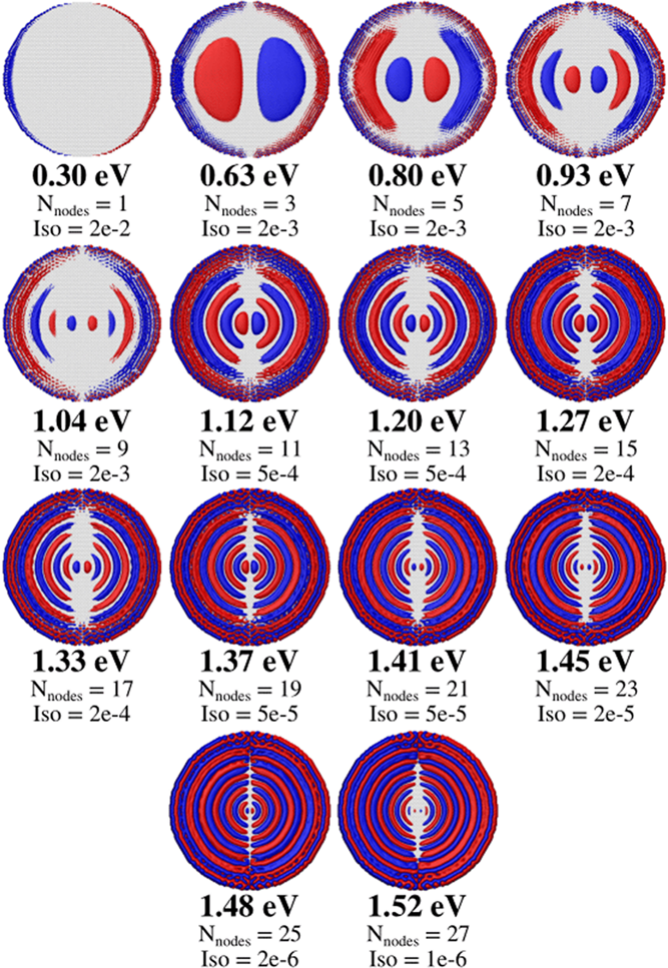
Graphical depiction of GD20 plasmon densities calculated at PRFs highlighted in Figure 2. The number of nodes (*N*_nodes_) and the isovalue for each plasmon mode are also reported. Densities are obtained by superimposing the Gaussian density associated with each ωFQ point charge. ωFQ charges have been calculated through the GMRES algorithm by setting ε_F_ = 1.51 eV, τ = 170 a.u., and *T* = 10^–6^.

Similar to σ_*X*_, the NI plot is characterized by a distribution of local maxima, with the same intrinsic error of PRFs. From an inspection of Figure 2, a strong correlation between the two sets of local maximum points is observed, and this is especially true for the most significant maxima highlighted in Figure 2. This result is not surprising: from eq 26, it is expected that a local maximum of σ_*X*_(ω) is necessarily associated with a local maximum (in absolute value) of ωFQ point charges. The charge densities associated with σ_*X*_(ω) highlighted local maxima are plotted in Figure 3, and they clearly represent plasmon modes of increasing order. In fact, the number of nodes (*N*_nodes_) is always odd for symmetry reasons and increases as frequency increases.^32^ Since the iterative procedure starts from the **q**_0_ = 0 vector, when the distance between the guess and the solution vectors increases, NI increases.

Moving to the global trend of the NI (see Figure 2), the required number of iterations is small for the first PRF at ω = 0.3 eV, and then the NI increases in the middle region of the spectrum and finally decreases. Two underlying mechanisms may explain this peculiar trend. First, the lowest-order plasmon modes (e.g., the dipolar one at 0.3 eV) are strongly localized on the edge of the system (see Figure 3). Therefore, a small number of large-valued point charges are involved in the excitation, but most charges are instead close to zero (e.g., those placed in the middle of the structure). By this, the guess vector **q**_0_ = 0 a.u. is a satisfactory starting point for the iterative procedure, and a small number of iterations are sufficient to obtain the solution vector. This is not true for the highest-order plasmon modes, which are instead delocalized all over the system (see Figure 3). In addition, the plasmonic response intensity (i.e., the point charges absolute value) strongly decreases when the excitation order increases (see Figure 2, top) because the number of nodes in the plasmon mode is larger. This is also evinced by the isovalue used to plot the densities in Figure 3, which decreases as the PRF increases. Therefore, the NI in correspondence to the highest-order plasmon modes tends to decrease. The presence of low-amplitude local maxima, represented by dashed lines in Figure 2, top panel, can be attributed to numerical artifacts (see Figure S11 in the SI).

**Figure 4:**
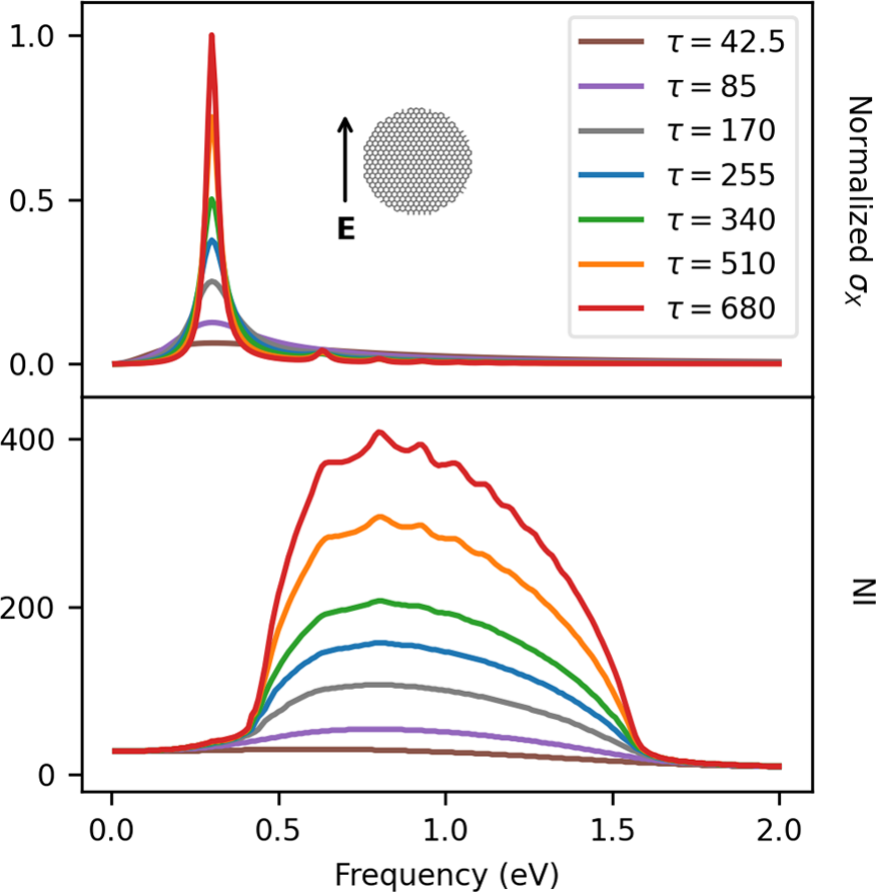
GD20 σ_*X*_ (ω) (top) and NI (bottom) as a function of τ (given in a.u.).

**Figure 5:**
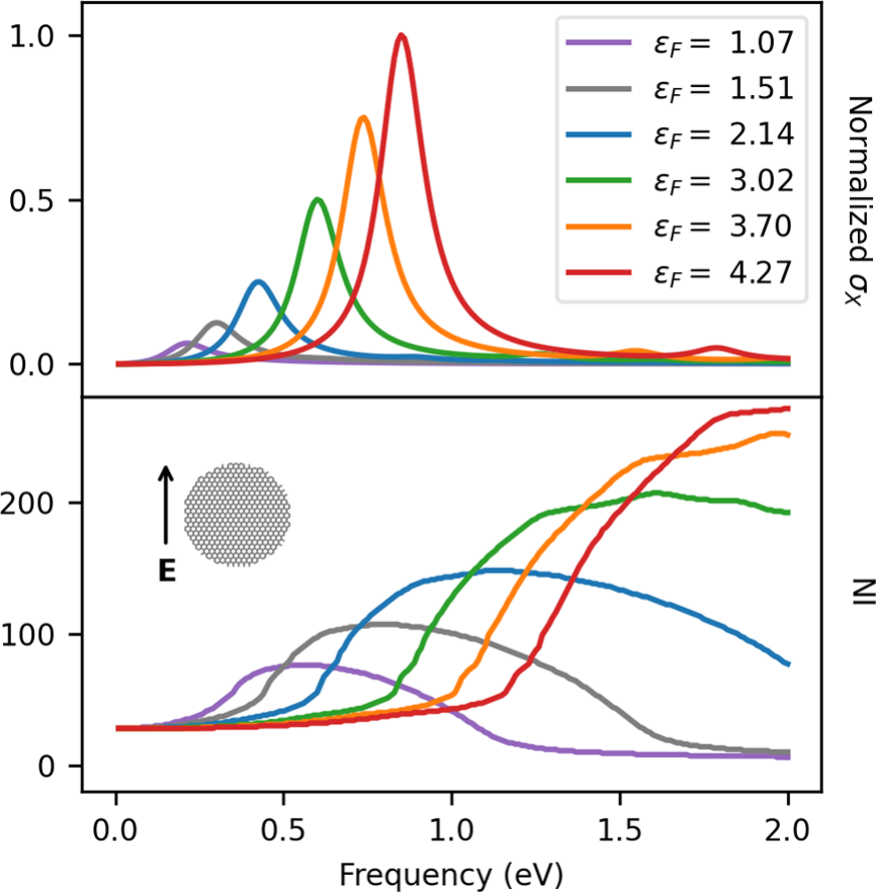
GD20 σ_*X*_ (top) and NI (bottom) as a function of the Fermi energy ε_F_ (given in eV).

The dependence of NI and σ_*X*_ on τ and ε_F_ is reported in Figures 4 and 5, respectively.

Focusing on the dependence on τ (Figure 4), we see that the PRF is not affected by this parameter, as it has been already reported in previous works.^29^ The main effect of the variation of τ is the shrinking of the excitation band shape and the associated increase of intensity. In the energy region between 0.5 and 1.5 eV, NI increases with τ, and new local maxima in both σ_*X*_ and NI at τ = 680 a.u. arise. These local maxima are associated with the high-order plasmon resonance modes identified in Figure 2 and represented in Figure 3.

**Figure 6:**
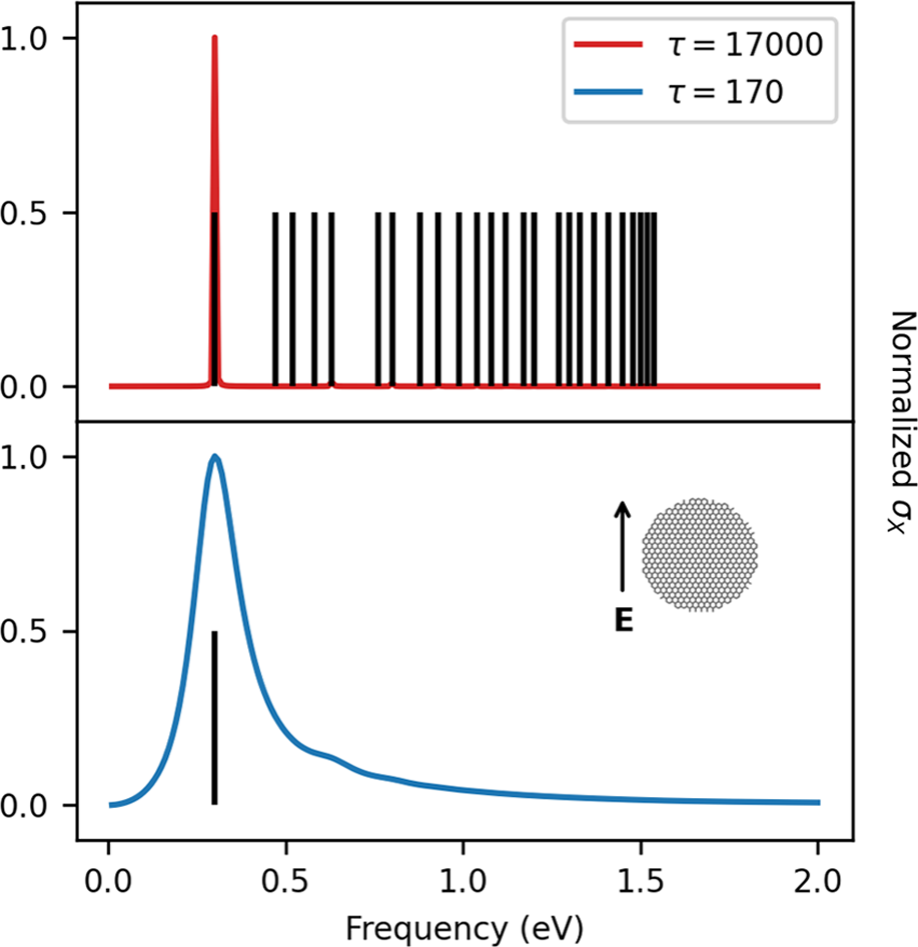
GD20 σ_*X*_ calculated by setting τ = 17 000 a.u. (top) or τ = 170 a.u. (bottom). Local maxima extrapolated from σ_*X*_ are reported as black sticks.

The most relevant plasmon resonance mode is the dipolar excitation because it is generally associated with the highest amplitude and the lowest PRF, which make it the most suitable for physical applications.^10^ A smaller value of τ can be adopted to achieve a reliable description of this excitation. In Figure 6, GD20 σ_*X*_ calculated by setting τ = 17 000 a.u. and τ = 170 a.u. and ε_F_ = 1.51 eV are reported. Clearly, the first plasmon excitation with PRF = 0.3 eV is the most intense for both τ values. For τ = 170 a.u., σ_*X*_ is characterized by a single maximum since the reduction of the relaxation time induces also a proportional reduction of the plasmon excitation intensities.^29^ By this, we can conclude that τ = 170 a.u. can be chosen to reduce the computational cost of the iterative procedure since we are mostly interested in the description of dipolar excitation.

The dependence of NI on ε_F_ is reported in Figure 5. We see that the increase of ε_F_ results in a blue shift of the PRF and in the increase of the absorption intensity. In fact, a higher value of ε_F_ is associated with an increase of *n*_2D_ (see eq 7) because a higher fraction of π electrons are involved in the excitation. The NI shows a similar trend because the global maximum is blue-shifted and the required number of iterations increases.

### 3.2 Computational Demand as a Function of the System’s Geometry

In this section, we investigate the dependence of the calculation’s convergence rate on the geometry of the system. The same CNT and GD structures investigated in the previous section have been selected, for which we have varied the characteristic dimensions (see Figure 1a,b).

#### 3.2.1 CNT

**Figure 7:**
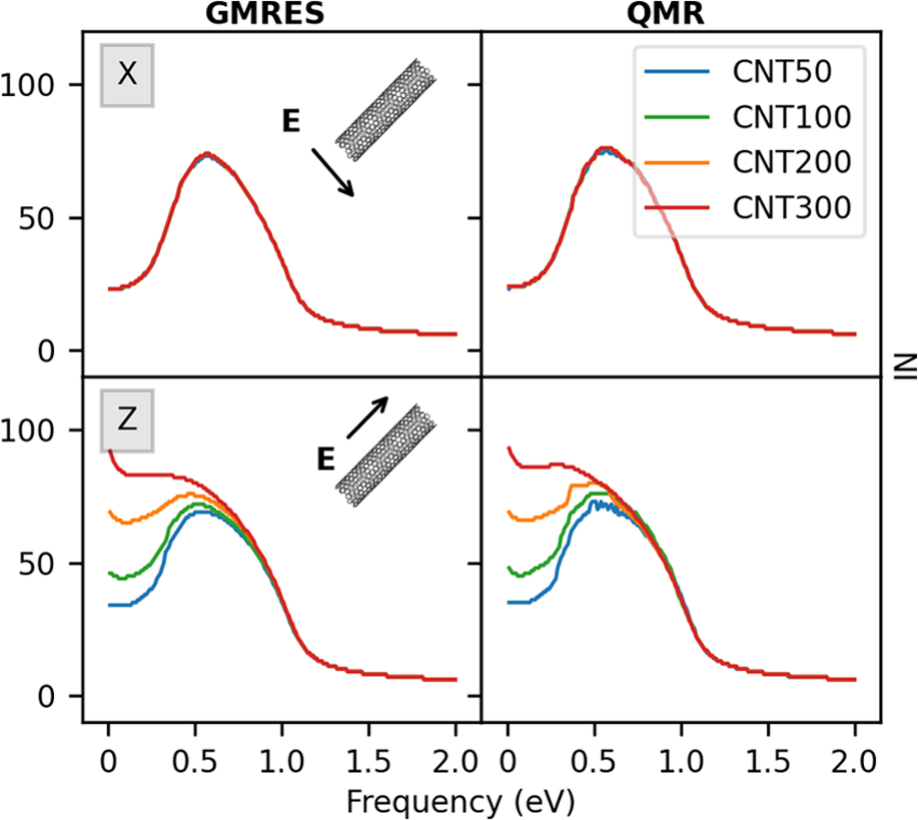
Convergence rate dependence on the CNT length *L* (see Table 1) as calculated by GMRES (left panel) and QMR (right panel). Both longitudinal (bottom) and transverse (top) polarizations of the external field are considered.

Table 1 reports the geometrical parameters of the selected structures. For each structure, the ωFQ linear system in eq 14 has been solved for 200 frequencies in the range between 0.01 and 2.0 eV with a constant step of 0.01 eV, by setting ε_F_ = 1.04 eV and τ = 170 a.u., respectively. First, we comment on the results obtained by fixing *d*_C_ = 1.36 nm and by varying *L* from 50 to 300 nm (see Table 1, top block). The data are shown in Figure 7 for both GMRES and QMR algorithms, in case the external field is aligned along the transverse (X) or longitudinal (Z) directions. We are then assuming that the two possible transverse directions (X and Y) provide the same polarization, even if all of the considered CNTs are chiral. In fact, the differences in the plasmonic response along the two directions are negligible (see Figure S12 in the SI). Figure 7, top panel, shows that the NI along the transverse direction is the same for all systems because the diameter is kept constant. In the case of longitudinal polarization, the number of iterative steps increases as the length of the system increases in the low-energy region of the spectrum, for both GMRES and QMR. This is due to the fact that PRFs are red-shifted as *L* increases, approaching 0 eV (see Table S2 in the SI), which is an expression of the so-called *lightning rod* effect.^55−57^ The *z*(ω) factor in eq 12 is therefore close to 0, and since the **A** matrix in eq 14 is singular, the number of iterations increases due to increased ill-conditioning.

**Table 1:** Geometrical Parameters of the Studied CNT Structures (see Figure 1a for Their Definition)^a^

	*L* (nm)	chiral numbers^b^	*d*_C_ (nm)	number of C atoms
CNT50	50	(8,12)	1.36	8208
CNT100	100			16 416
CNT200	200			32 832
CNT300	300			49 248
CNT1	50	(8,12)	1.36	8208
CNT2		(16,24)	2.77	16 416
CNT3		(24,36)	4.10	24 624
CNT4		(32,48)	5.46	32 832

a The number of atoms for each structure is also given.

b The relation between the diameter *d*_C_ and the chiral numbers (*n*, *m*) is , where *b*_G_ is the graphene lattice basis vector norm, i.e., 0.246 nm.^54^

**Figure 8:**
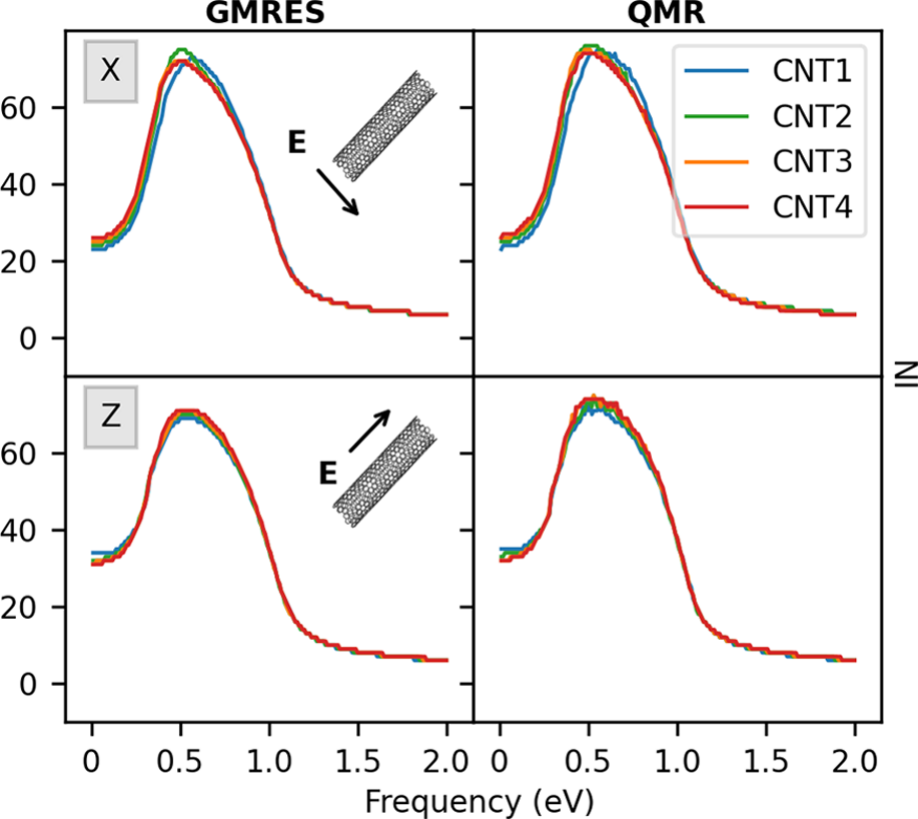
Convergence rate dependence on the CNT diameter *d*_C_ (see Table 1) as calculated by GMRES (left panel) and QMR (right panel). Both longitudinal (bottom) and transverse (top) polarizations of the external field are reported.

We now move to comment on the results obtained by varying the CNT *d*_C_, by keeping constant *L* = 50 nm (see Table 1, bottom block). Computed GMRES and QMR NI for such systems are reported in Figure 8. Differently from the previous case, the NI trend does not show a strong dependence on *d*_C_, for both transverse and longitudinal directions of the applied electric field. This is related to the fact that, although PRF energies are red-shifted as *d*_C_ increases (along the X direction), the smallest PRF associated with the dipolar plasmon is far from 0 eV (0.37 eV for CNT4, see Table S2 in the SI). Therefore, in this case, severe ill-conditioning is avoided and the NI remains almost constant.

As a final comment, we note that GMRES and QMR provide almost the same convergence rate. In particular, QMR NI is usually slightly higher than GMRES, thus confirming what has been observed in Section 5.1 (see also Figure S8 in the SI).

#### 3.2.2 GD

Let us now focus on the NI calculated for four different GDs, obtained by varying the *d*_D_ diameter (see Figure 1b). Geometrical parameters are reported in Table 2. The ωFQ linear systems have been solved by setting the same parameters exploited in the case of CNTs, and by imposing ε_F_ = 1.51 eV. In this case, due to symmetry reasons, the external electric field is polarized along one axis only.

**Table 2:** Geometrical Parameters for the Studied GDs (see Figure 1b for Their Definition)^a^

ID	*d*_D_ (nm)	number of C atoms
GD20	20	11 970
GD26	26	20 058
GD32	32	30 788
GD36	36	38 974

a The number of atoms for each structure is also given.

**Figure 9:**
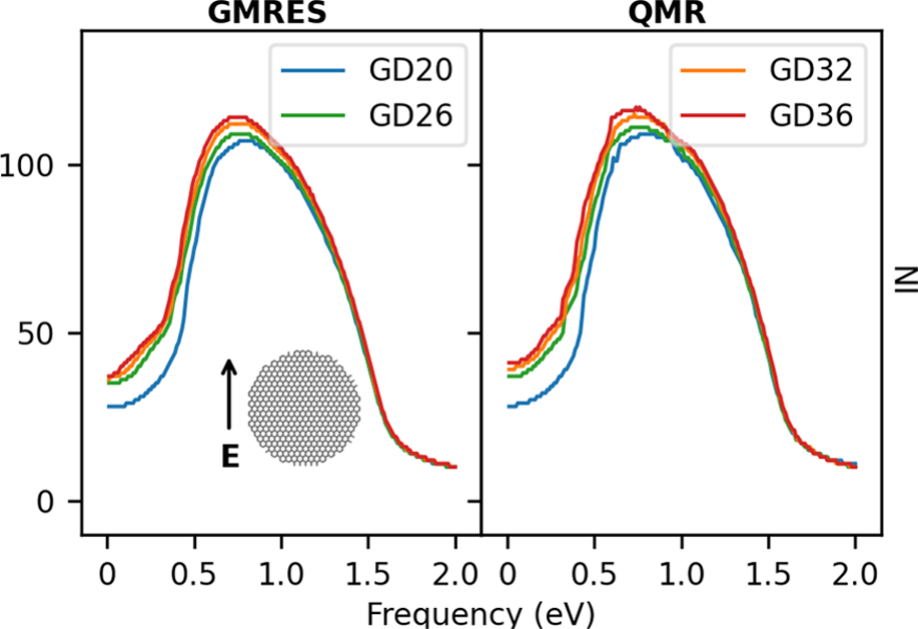
Convergence rate dependence on the GD diameter *d*_D_ (see Table 2), as calculated by using GMRES (left panel) and QMR (right panel).

For each structure, NI has been calculated for GMRES and QMR, and the results are reported in Figure 9. Interestingly, the NI presents a weak dependence on the *d*_D_ diameter. In fact, the largest difference in the number of iterations is about 10 between GD36 and GD20. However, GD36 has almost four times the atoms of GD20, thus demonstrating the favorable scalability of the two algorithms. We finally note that also in this case the PRFs are red-shifted as the size of the system increases (see Table S2 in the SI).

### 3.3 Modeling plasmonic properties of real-size systems

To finally demonstrate the robustness of the developed iterative methods to solve the ωFQ linear system, we investigated the plasmonic response of real-size systems, composed of roughly one million atoms. When dealing with large-sized structures, two main issues arise. From the theoretical point of view, the quasi-static approximation on which ωFQ equations are based could be no longer valid. From the technical point of view, when the number of atoms increases, the storage of the ωFQ matrix in physical memory can rapidly become unfeasible. Such a problem can be handled by adopting a matrix-free version of the GMRES algorithm, where the **A** matrix in eq 14 is not explicitly built. In fact, the iterative algorithm only requires calculating the matrix–vector product **Ax**, which can be performed on the fly during the execution of the program. This means that at each iterative step *k*, the new Krylov basis vector is obtained as
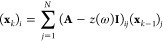
27where the element (*i*, *j*) of the matrix **A** – *z*(ω) **I** is calculated when required by the algorithm. On the other hand, each matrix element has to be calculated from scratch; therefore, the on-the-fly version of GMRES would require larger computational time, without affecting the number of iterations. Nevertheless, the on-the-fly matrix–vector product can be efficiently calculated in a parallel environment and memory requirements are negligible with respect to the standard GMRES procedure because only the iterative vectors should be kept in memory during the solution procedure.

To showcase the performance of GMRES when applied to large systems, we have selected three structures composed of roughly 1 million atoms: a carbon nanotube −CNT1M–, a graphene disk −GD1M–, and a sodium nanorod −NR1M– (see Table S11 given in the SI, for geometrical parameters). The latter is genuinely different from the other two structures and has been selected to further demonstrate the reliability of the method to study the optical properties of metal nanostructures.

For each of the constructed structures, the longitudinal absorption cross sections and the NI have been calculated. It has been shown in Section 5.2 that the convergence criterion based on the 2-norm of the residual vector (see eq 25) is not size-independent. The root-mean-squared error (RMSE) (see eq 35) has been computed at each iterative step, and it has been compared with a threshold *T*, to check the convergence. In particular, we have set *T* = 10^–5^, which according to Section 5.2 is a good and size-independent compromise between accuracy and computational cost.

#### 3.3.1 CNT1M

**Figure 10:**
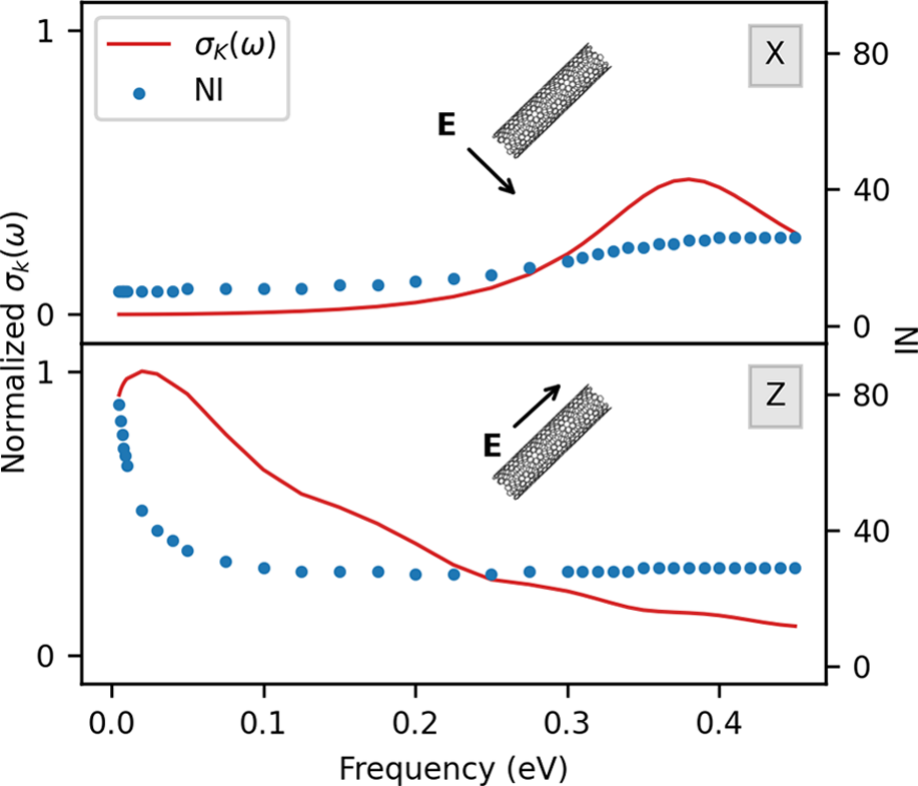
CNT1M σ_*k*_(ω) (solid red line) and NI (blue dots). Both longitudinal (*k* = *Z*, bottom) and transverse (*k* = *X*, top) polarizations of the external field are reported. GMRES algorithm: RMSE = 10^–5^.

The calculations on CNT1M have been performed by applying an external field along the transverse and longitudinal directions, at 35 different frequencies in the range between 0.005 and 0.45 eV, by setting τ = 170 a.u. and ε_F_ = 1.03 eV. The longitudinal absorption cross sections and the NI are reported in Figure 10. The transverse PRF is placed at about 0.38 eV, which is close to the value for CNT4, which has the same diameter (see Table 1). The longitudinal PRF is instead placed at 0.02 eV, which is smaller than the value for CNT300, which has a length of 300 nm. This is not surprising because the PRF is red-shifted as the length of the system increases. The required number of iterations as a function of the external field frequency is reported in Figure 10. We note that the maximum number of iterations is 80, which is lower than what we have obtained for CNT300 (see Section 3.2). This is due to the larger convergence threshold chosen for the iterative procedure; overall, a mean value of about 30 iterations is sufficient to reach the convergence. Since the number of iterations is modest, restarted GMRES has not been considered for such large systems. Moving on to discuss the computational time, our implementation permits to calculate about 4 matrix–vector products per hour, thus resulting in a total time of about 487 h.

#### 3.3.2 GD1M

**Figure 11:**
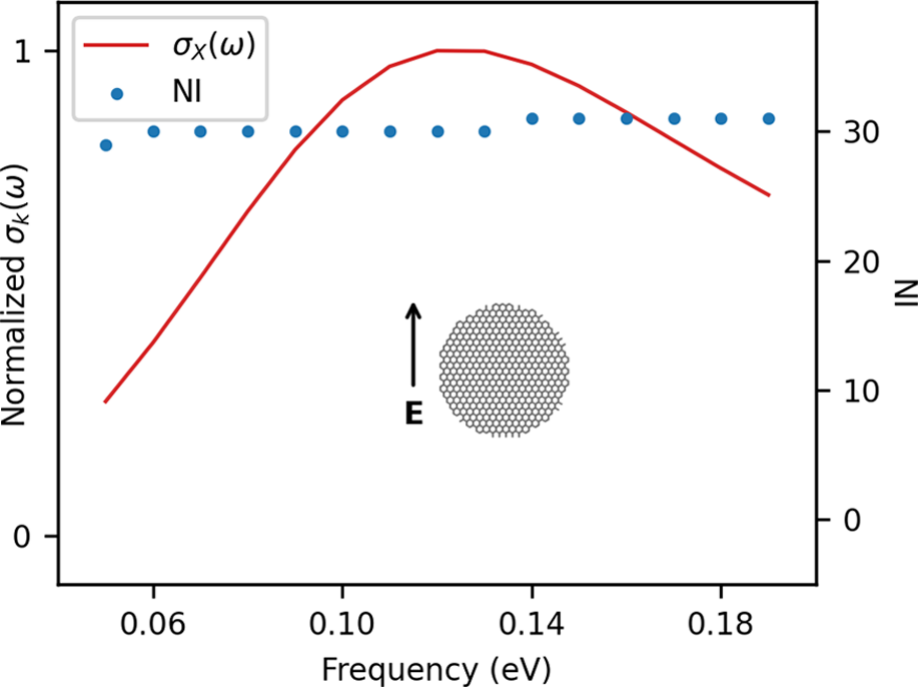
GD1M σ_*X*_(ω) (solid red line) and NI (blue dots). GMRES algorithm: RMSE = 10^–5^.

The ωFQ linear system has been solved for GD1M with an external field polarization vector lying on the GD plane (*X*), at 15 different frequencies in the range between 0.05 and 0.19 eV with a constant step of 0.01 eV. We set τ = 170 a.u. and ε_F_ = 1.84 eV. The computed σ_*X*_ and NI are reported in Figure 11. The transverse dipolar PRF for this system occurs at 0.12 eV, which is smaller than the values for the smallest GDs studied in the previous sections. The required number of iterations to reach convergence is about 30 for each external field frequency, i.e., smaller than what is required by GDs described in Section 3.2. As it has been stated for CNT, this is mainly due to the setting of a larger RMSE threshold.

#### 3.3.3 NR1M

**Figure 12:**
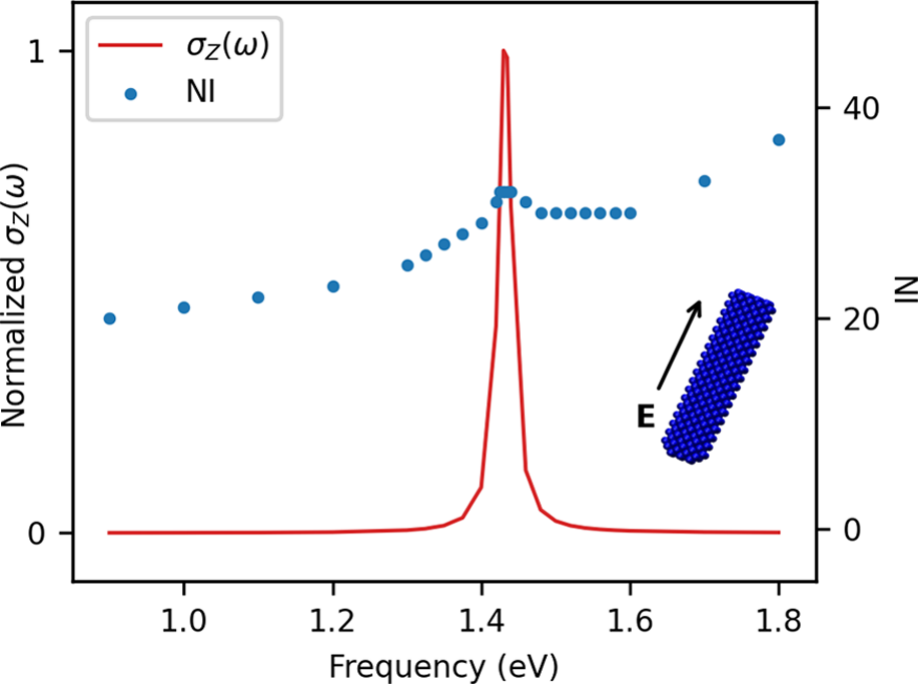
NR1M longitudinal σ_*Z*_(ω) (solid red line) and NI (blue dots). GMRES algorithm: RMSE = 10^–5^.

Finally, we have calculated the plasmonic response of the NR1M system. This is a 3D nanostructure; therefore, a general expression of the **K**^tot^ matrix in terms of the 3D electron density *n*_0_ (see eq 5) needs to be exploited. All calculations have been performed with ωFQ parameters for sodium reported in previous work.^28^ The linear system in eq 14 has been solved for 24 frequencies in the range between 0.9 and 1.8 eV (unevenly distributed) with an external field aligned along the longitudinal (*Z*) direction. As for CNT1M and GD1M, the RMSE threshold was set to 10^–5^. The computed σ_*Z*_ and the corresponding NI are reported in Figure 12.

The longitudinal PRF is placed at about 1.43 eV, which is blue-shifted with respect to the longitudinal PRF of CNT1M, due to the fact that the electronic properties of the two materials are different. As for the previously studied carbon-based system, the value of the longitudinal PRF is red-shifted with respect to smaller sodium nanorods reported in previous work.^28^ This is once again due to the lighting rod effect discussed above. Similar to previous cases, a mean value of about 30 iterations is sufficient to reach convergence.

#### Accounting for Structural Defects

As the last example, we focus on a peculiar feature of ωFQ, i.e., its ability to model nanostructures with structural defects. These studies are possible due to the atomistic nature of our model; in fact, such kind of information cannot clearly be extracted by modeling the structure by means of continuum approaches.

**Figure 13:**
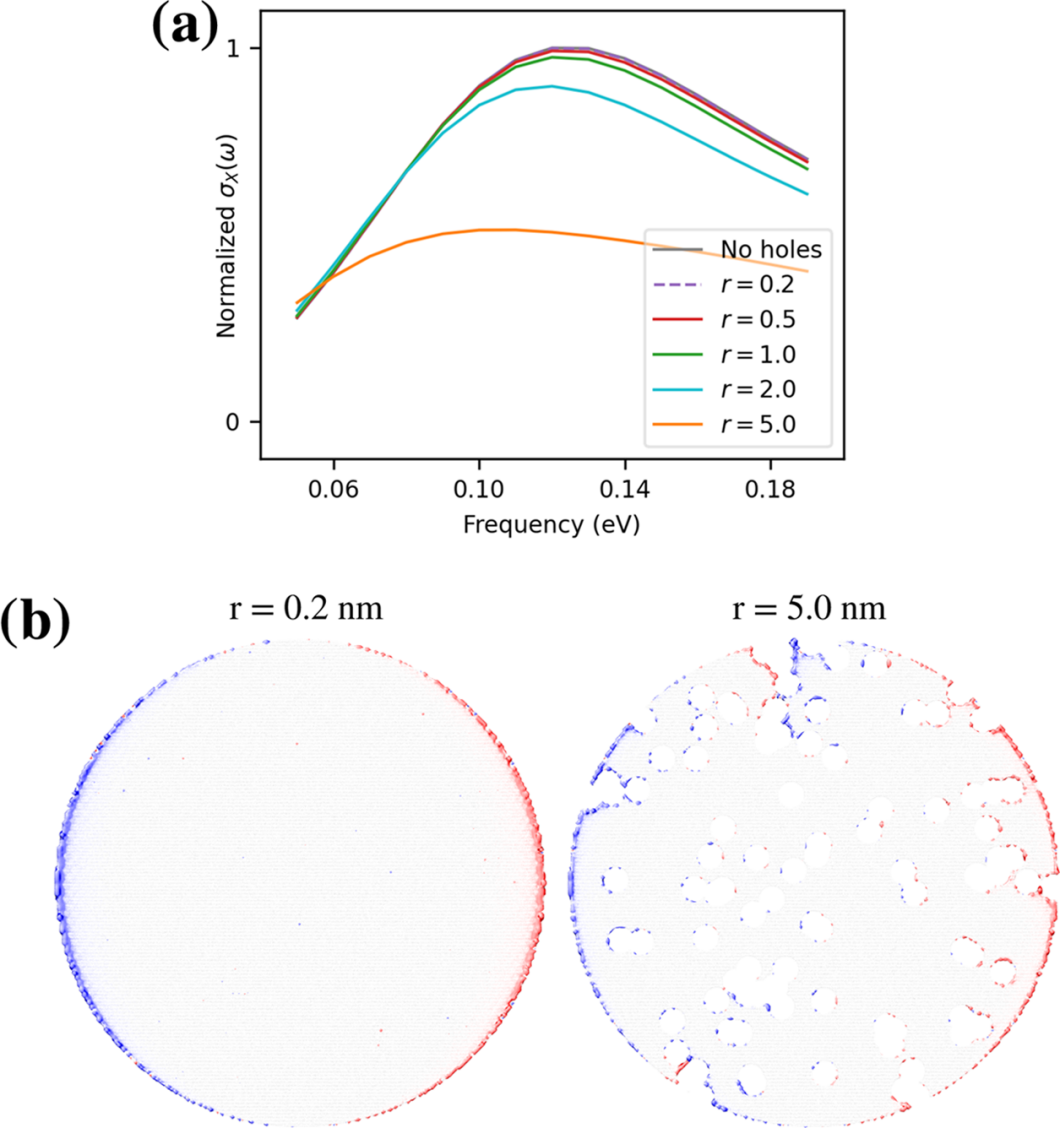
(a) GD1M σ_*X*_(ω) as a function of hole radius (*r*, in nm). GMRES algorithm: RMSE = 10^–5^. (b) Graphical depiction of the plasmon density for *r* = 0.2 nm (left) and *r* = 5.0 nm (right). Isovalue: 0.005.

In Figure 13a and b, ωFQ is applied to a set of graphene disks, which have been created by introducing 100 holes with radius *r* in the GD1M system and in particular by removing all carbon atoms within a distance smaller than *r* from a given carbon atom. The hole radius *r* has been set to five different values in the range between 0.2 and 5 nm. For each of these structures, the ωFQ linear system has been solved with an external field polarized along the *X*-axis, at 15 different frequencies in the range between 0.05 and 0.19 eV with a constant step of 0.01 eV. The relaxation time (τ) has been set to 170 a.u., while the Fermi energy has been adjusted so to impose the same electron density of the defect-free GD1M structure (see eq 8). Note that our model can be applied to any kind (and number) of defects; here, we show this particular case as a proof of concept.

The absorption cross sections along the *X* direction for each structure are reported in Figure 13a. The results are strongly affected by the value of the radius of the holes: in fact, by increasing it, the absorption maximum red-shifts and its intensity decreases. The red shift may be explained by focusing on the plasmon density calculated at the plasmon resonance frequency (see Figure 13b). In fact, when the radius of the holes is small (*r* = 0.2 nm), the plasmon density retains a dipolar nature. By contrast, for larger radii (*r* = 5.0 nm), the overall dipolar nature of the plasmon is no more evident because other local dipoles appear on the edges of each hole. Also, in the structures with the largest holes, more and more atoms are removed from the initial structure. As a consequence, the intensity of the absorption peak decreases by increasing the radius of the defects.

## 4 Conclusions

In this paper, we have discussed how to substantially increase the applicability of classical, fully atomistic approaches to the calculation of the plasmonic properties of real-size nanostructures (carbon nanotubes, graphene-based materials, and metal nanoparticles). In particular, we have proposed a unified model, specified for the recently developed fully atomistic ωFQ approach, which is able to accurately describe both 2D and 3D systems with similar accuracy. By taking advantage of the new formalism, the existence and uniqueness of the ωFQ solution have been demonstrated, allowing for the investigation of the performances of different state-of-the-art numerical techniques to reach the solution. The novel formulation also permits a significant decrease in computational demand. Thus, the calculation of the plasmonic properties of structures constituted by 1 million atoms has become possible, remarkably also for systems with structural defects.

The implemented iterative procedures are characterized by three main bottlenecks, and possible solutions will be investigated in future work. On the one hand, the number of matrix–vector products needed to build approximate solutions to the linear system is associated with the computational complexity of *O*(*N*^2^). Linear scaling in matrix–vector products might be achieved through the fast multipole method (FMM),^58−61^ a numerical technique that can be adopted to build an approximation to the long-range electrostatic forces, which has already been applied to plasmonic substrates.^16^ In addition, we have tested different preconditioning strategies of the ωFQ linear system^62−64^ without obtaining a significant improvement in terms of both memory requirements and computational time. Such an extension will allow affording systems even larger than those studied in this work. However, in this case, the quasi-static approximation on which ωFQ relies may be no longer valid. Therefore, retardation effects would need to be included in the model, similar to what has already been proposed for continuum approaches.^26,65^

Finally, to study the plasmonic properties of a given nanostructure along a specific spectral region, the ωFQ linear system can be solved independently for each frequency. However, a change in frequency only affects the uniform diagonal shift of the coefficient matrix in eq 14. Therefore, the shift-invariance property of the Krylov subspaces may be exploited by resorting to the so-called subspace recycling techniques.^66,67^

To conclude, and to give the reader a further roadmap for the development and application of ωFQ, it appears to have high potentialities to describe surface-enhanced spectroscopies, either based on graphene-based substrates or metal nanoparticles.^10,68^ To this end, ωFQ needs to be coupled with a quantum Hamiltonian describing the adsorbed molecules, in a QM/MM fashion.^13−17^ Also, to accurately describe d-electron metals, such as silver and gold, ωFQ might need to be extended to treat interband contributions, e.g., by adding atomic polarizabilities similar to what is done in QM/MM approaches.^36,69,70^

## Figures and Tables

**Figure 14:**
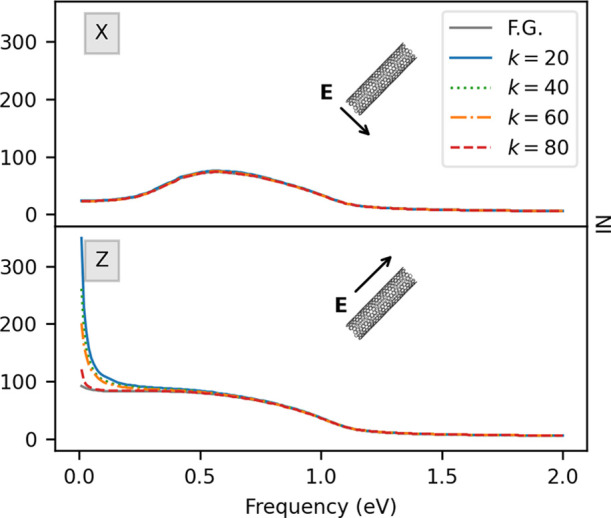
CNT300 NI dependence on the dimension of the Krylov subspace *k* as calculated by (*k*). The full GMRES (F.G.) values are also shown. Both longitudinal (bottom) and transverse (top) external fields are considered.

**Figure 15:**
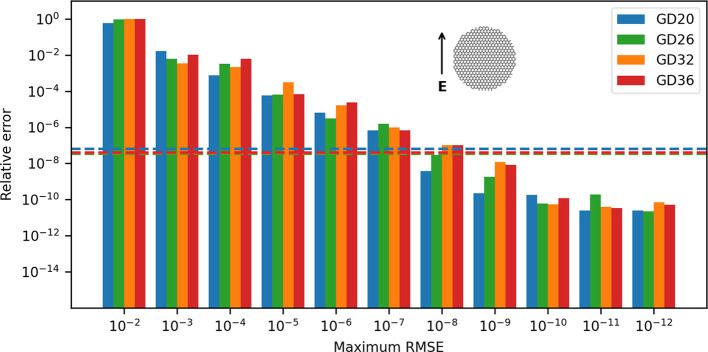
Average relative errors for GD20, GD26, GD32, and GD36 (see Table 1) of the GMRES iterative solution with respect to LU factorization of the coefficient matrix at different choices of the RMSE introduced in eq 35. Dashed lines indicate an approximate upper bound of the intrinsic precision associated with the inversion algorithm.

**Figure 16:**
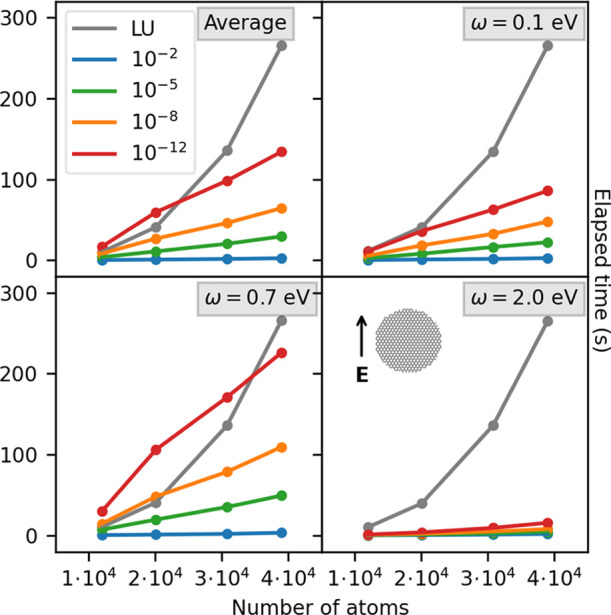
Computational time required to solve the ωFQ linear system as a function of the number of atoms in GD structures. Average (top, left), ω = 0.1 eV (top, right), ω = 0.7 eV (bottom, left), and ω = 2.0 eV (bottom, right).

## Appendix

### 5.1 Solution strategies

One of the most powerful techniques to solve a linear system **Ax** = **b** (with a generic matrix **A**) of order *N* is to resort to Krylov subspace iterative methods.^71,72^ The idea behind this family of methods is to build an approximate solution to the linear system at step *m* in the *m*-dimensional affine subspace , where  is the Krylov subspace defined as

28

where **r**_0_ is the residual associated with the initial guess **x**_0_.

There are many algorithms that fall into the Krylov methods family, and different taxonomies have been proposed.^73−75^ Such methods are exploited to solve large linear systems arising in different fields of computational chemistry.^21,23,76,77^

In this work, two different Krylov-based iterative methods have been tested for the solution of eq 14, namely, the Generalized Minimum RESidual algorithm (GMRES)^78^ and the quasi-minimal residual (QMR) method.^79^

#### 5.1.1 GMRES

It is a general approach to nonsymmetric linear systems.^78^ At each step *m*, an approximate solution of the linear system is obtained as

29

where **V**_*m*_ is an orthonormal basis of the *m*-dimensional Krylov space . The vector **y**_*m*_ is determined such that the 2-norm of the residual **r**_*m*_ = **b** – **Ax**_*m*_ is minimal over . The orthonormal basis of  is obtained via the Arnoldi process,^80^ and the orthogonal projection of **A** onto  leads to an upper Hessenberg matrix **H**_*m*_ = **V**_*m*_^†^**AV**_*m*_.^81^ Therefore, the least-squares problem can be efficiently solved through QR factorization of **H**_*m*_.^81^ The QR decomposition of **H**_*m*_ can be updated cheaply on each iteration, but at each step, a new vector must be stored, so the memory cost is not constant during the iterative procedure.^81^

To reduce the memory required by GMRES, the so-called “restarted” GMRES algorithm has been developed, also known as GMRES(*k*).^78,82^ There, the iterative procedure is stopped after *k* steps, and the GMRES algorithm is restarted using the last iterative vector **x**_*k*_ as the new initial guess vector from which the Krylov subspace is built once again. By this, no more than *k* vectors are stored in memory at the same time; however, the algorithm is expected to converge more slowly than standard GMRES.^82^

#### 5.1.2 QMR

Similar to GMRES, QMR has been developed for solving nonsymmetric linear systems. In this case, the coupled two-term Lanczos algorithm is adopted to generate two Krylov spaces  and , where **p**_0_ and **q**_0_ are two initial vectors.^83^ At each step, an approximate solution to the complex-valued linear system is defined as

30

which is similar to what is done by GMRES (see eq 29). In fact, **P**_*m*_ is the basis set of the Krylov space , while the **y**_*m*_ vector is associated with an approximate 2-norm of the residual.^83^ In the QMR formalism, the residual **r**_*m*_ = **b** – **Ax**_*m*_ can be written as

31

where the matrices **M**_*m*_ and **N**_*m*_ and the vector **f**_*m*_ are obtained through the basis sets previously defined.^83^ In QMR, the vector **y**_*m*_ is chosen to minimize the quantity in parenthesis in eq 31. In other words, **y**_*m*_ is the solution of the least-squares problem min_**y**_*m*__∥**f**_*m*_ – *N*_*m*_**y**_*m*_∥. Such a procedure yields a “quasi-minimization” of the residual; therefore, it is expected to converge more slowly than GMRES. On the other hand, at each step, a fixed number of vectors need to be calculated; thus, memory requirements are constant along the whole iterative procedure.

Note that we have considered the QMR algorithm because a simplified version has been proposed by Freund and Nachtigal in the case of **J**-symmetric coefficient matrices, i.e., such that **A**^*T*^**J** = **JA** for an SPD matrix **J**.^51^ This property, which we have demonstrated for the ωFQ matrix in Section 2.1 (with **J** = **D**), allows us to simplify the Lanczos process by choosing the starting vectors such that **q**_0_ = **Jp**_0_. In this way, the computational effort to compute the basis sets of the involved Krylov space is reduced because the calculation of the matrix–vector product **A**^*T*^**x** is not needed. As a final remark, in this work, the Krylov iterative methods have been applied without resorting to preconditioning techniques (see Section 2.2).

### 5.2 Dependence of the Convergence Rate on Algorithm Parameters

We now move to consider the technical parameters associated with the iterative procedure and how they affect the convergence rate. We first study the performance of GMRES(*k*) (see Section 5.1), by taking as a reference system the CNT300 structure (see Table 2) and exploiting the same parameters used above for CNTs for solving the ωFQ linear system. The iterative procedure has been performed by varying *k* (between 20 and 80) and by keeping the threshold fixed to *T* = 10^–6^. Computed NI are reported in Figure 14, together with the corresponding results obtained with the full GMRES (F.G.) algorithm (i.e., nonrestarted). For X and Y polarizations, the reduction of the Krylov subspace does not affect the NI behavior. By contrast, for Z polarization, GMRES(*k*) and F.G. procedures yield different NI trends in the region between 0 and 0.2 eV. In fact, GMRES(*k*) requires a larger number of iterations than F.G. version to reach convergence, independently of the dimension of the Krylov subspace *k*. This is once again due to singularities arising when PRF approaches 0 eV, which yield ill-conditioning that is exacerbated in the restarted version of the algorithm. Such an explanation is corroborated by the evidence that the number of iterations required to reach convergence decreases by increasing the dimension of the Krylov subspace *k*. For each *k*, we also notice that GMRES(*k*) is almost as efficient as the F.G. procedure in the remaining part of the spectrum. Therefore, in case the PRF is far from 0 eV, the same results can be obtained by a cheaper iterative procedure in terms of memory requirements because a smaller number of Krylov basis vectors have to be stored to build up the solution vector.

Another quantity that can strongly affect the NI is the threshold *T* defined in eq 25, which in all previous calculations has been fixed to 10^–6^. We notice however that *T* is independent of the size of the system. Therefore, we can expect that the mean precision of the iterative solution, averaged for each charge, is higher when the number of atoms increases. In fact, eq 25 can be rewritten as

32

If we assume that the absolute error is the same for each point charge, i.e., *R*_*i*_ – ((**A** – z(ω)**I**)**q**_*k*_)_*i*_ = δ*q*, and we plug this approximation in eq 32, we obtain

33

Therefore, for a given threshold *T*, the absolute error on each charge decreases when the number of atoms *N* increases.

To obtain a size-independent estimate of the accuracy of the iterative solution over all of the ωFQ charges, we can introduce the following definition of the root-mean-squared error (RMSE)

34

Then, we can define a new convergence criterion as

35

We can now investigate the accuracy of the iterative solution by adopting different RMSE thresholds. As a precision measure, we consider the longitudinal absorption cross section σ_*X*_ calculated for a set of GDs (GD20, GD26, GD32, GD36 in Table 2) applying an electric field with a polarization vector lying on the molecular plane. The linear system has been solved with both GMRES and a direct procedure, i.e., an LU factorization of the coefficient matrix.^81^ For each selected RMSE value, the σ_*X*_ relative error between GMRES and LU factorization averaged over all of the considered frequencies (0.0–2.0 eV, with a step of 0.1 eV) has been calculated, and the results are graphically depicted in Figure 15. An approximate upper bound of the intrinsic precision associated with the factorization algorithm is also plotted (see Section S3 in the SI).

It can be seen that the accuracy of the iterative solution for the different systems is almost constant for a specific RMSE value. In particular, by imposing RMSE ≤ 10^–4^, the correct order of magnitude obtained by the LU solution can be recovered by the iterative procedure, i.e., the relative error is ≤10^–1^. To further demonstrate that the RMSE criterion is effectively size-independent, we performed the same analysis discussed above also for the CNT case (see Figure S15 in the SI).

We finally move to discuss the computational time required by the different choices of the RMSE value. In particular, such an analysis has been performed on the four GDs reported in Table 2. The computational time required to solve the ωFQ linear system for 20 frequencies in the range between 0.0 and 2.0 eV with a step of 0.1 eV is given in Figure 16 (raw data can be found in Tables S3–S6 in the SI). Both direct solution (LU factorization, see Section S2 in the SI) and the full GMRES algorithm with different choices of RMSE have been considered. Notice that we are showing the computer time required by the solution of the ωFQ linear system. This means that the coefficient matrix **A** (eq 11) and right-hand side (eq 13) construction are not taken into account to allow for a direct comparison with the factorization algorithm. All calculations have been performed on a Xeon Gold 5120 (56 cores, 2.2 GHz) cluster node equipped with 256 GB RAM.

In Figure 16, the average computational time is reported (top, left panel) together with the time required to solve the ωFQ linear system for three selected frequencies, i.e., 0.1 (lower bound) and 0.7 and 2.0 eV (upper bound). ω = 0.7 eV has been chosen because it corresponds to the maximum number of iterations required by full GMRES to converge (see also Figure 9). Figure 16 clearly shows that the direct solution through LU factorization and the GMRES iterative procedure intrinsically differ in terms of scaling with the dimension of the linear system (i.e., the number of atoms). In fact, the former has a complexity of *O*(*N*^3^), while the latter scales as *O*(*N*^2^). Such a difference is highlighted by the computational times in Figure 16: the increase of the computational time for the direct solution (gray line) is larger with respect to the iterative procedure when the number of atoms increases, independently of the RMSE value exploited in the GMRES algorithm. Also, we note that, differently from the LU-based algorithm, the computational time required by the iterative method is not constant across the frequency range. This can be explained by the fact that the number of iterations required to converge to the solution depends on the external frequency (see also Figure 9). Finally, we remark that even at 0.7 eV GMRES is more efficient than the inversion algorithm. In particular, a good compromise between accuracy and computational efficiency can be reached by using RMSE = 10^–5^, for which the computational time of the inversion solution can be reduced by a factor of 10 for the largest studied structure.

The computational time analysis has been performed also for the QMR algorithm (see Section S2 and Tables S7–S10 in the SI). From an inspection of the numerical results, it emerges that, for a given RMSE, QMR requires roughly twice the computational time than GMRES. In fact, as it has been shown above, GMRES and QMR need a similar number of iterations to converge; however, for each iteration, GMRES performs a single matrix–vector product, while QMR computes two matrix–vector products (one with the coefficient matrix, and one with the **D** matrix defined in eq 3). Therefore, although the computational and memory costs of GMRES are not fixed during the iterative procedure, it outperforms QMR because of the lower number of matrix–vector products that are needed to build the Krylov subspace.

## Supporting Information Available

Preconditioning of the ωFQ linear system; GD26 absorption cross section with and without dangling bonds; GD20 absorption cross section with τ = 17 000 a.u. GD20 plasmon densities; CNT50 transverse absorption cross sections; PRFs of Tables 1 and 2; approximate upper bound derivation of the precision of the factorization algorithm; computational timings of GD20 for iterative and factorization algorithm; relative errors of GMRES solution for CNT50, CNT100, CNT150, and CNT200; raw data of computational timings for systems in Table 2 for GMRES and QMR algorithms; and geometrical parameters of CNT1M, GD1M, and NR1M (PDF)
