# Correction to “Going Beyond the Limits of Classical Atomistic Modeling of Plasmonic Nanostructures”

**DOI:** 10.1021/acs.jpcc.3c01565

**Published:** 2023-03-21

**Authors:** Piero Lafiosca, Tommaso Giovannini, Michele Benzi, Chiara Cappelli

In the original paper, the Fermi energy values ε_*F*_ are incorrect. This affects the data in Figure 5 and the discussion of numerical results. As reported in ref 29, the Fermi energy can be computed from *n*_2D_, i.e., graphene 2D electron density (see eq 8 in the original paper) by exploiting the following equation:
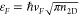
1where ℏ is the reduced Planck constant and *v*_*F*_ is the Fermi velocity. The 3D atomic effective electron density *n*_0_ can be computed as
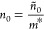
2where *ñ*_0_ is the 3D atomic electron density and m* is the effective electron mass, which in the case of metallic nanostructures is usually approximated to 1. However, for graphene-based materials, *n*_0_ can be obtained from the 2D electron density by assuming that (see ref 29 in the original paper):
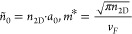
where *a*_0_ is the Bohr radius. By replacing such definitions in eq 2 we obtain
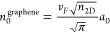
3In our original paper, we have erroneously computed the Fermi energy ε_*F*_ in eq 1 by using the 3D effective electron density *n*_0_^graphene^ instead of the 2D electron density *n*_2D_. Since ε_*F*_ never explicitly enters the equations but is only a computed quantity, all numerical results and the discussion of the numerical performance of the iterative solution are unaffected. The correct version of Figure 5 is reported in the following Figure 1, while the corrections that need to be applied to the values reported in the original paper are summarized in Table 1.

**Figure 1:**
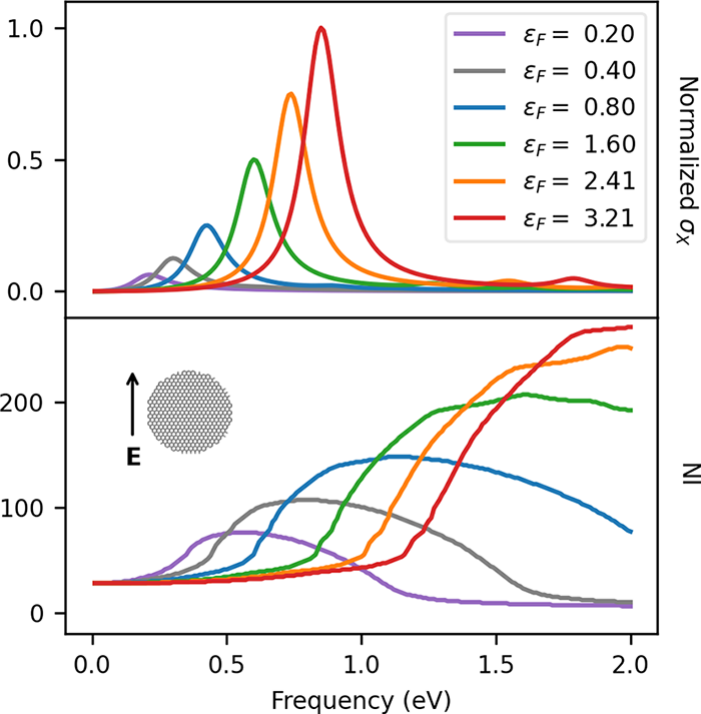
GD20 σ_*X*_ (top) and NI (bottom) as a function of the Fermi energy ε_*F*_ (given in eV).

**Table 1:** Numerical Values (Old and New) of ε_*F*_, with the Indication of the Associated Graphene-Based System^a^

Position	System	Old ε_*F*_ (eV)	New ε_*F*_ (eV)
p. 23852, right column	GD20	1.51	0.40
p. 23853, caption of Figure 3	GD20	1.51	0.40
p. 23854, left column	GD20	1.51	0.40
p. 23854, right column	CNTs	1.04	0.19
p. 23855, right column	GDs	1.51	0.40
p. 23856, left column	CNT1M	1.03	0.19
p. 23856, right column	GD1M	1.84	0.60
p. S4, Supporting Information	GD36	1.51	0.40

a The same notation as the original text is used. All data are reported in eV.

