# Supplementary material for: Going Beyond the Limits of Classical Atomistic Modeling of Plasmonic Nanostructures
Source: J Phys Chem C Nanomater Interfaces. 2021 Oct 26;125(43):23848–63. doi: 10.1021/acs.jpcc.1c04716 (PMC8573767; doi:10.1021/acs.jpcc.1c04716)
Supplement: Supplementary file 1 — jp1c04716_si_001.pdf [file jp1c04716_si_001.pdf]

Supporting Information: Going Beyond the Limits of Classical Atomistic Modeling of Plasmonic Nanostructures

Piero Lafiosca, Tommaso Giovannini, Michele Benzi, and Chiara Cappelli*

Scuola Normale Superiore, Piazza dei Cavalieri 7, 56126 Pisa, Italy.

E-mail: chiara.cappelli@sns.it

S1 Preconditioning of the linear system

Given the ωFQ linear system:

$$(\mathbf{A} - z(\omega)\mathbf{I})\mathbf{q} = \mathbf{R}, \tag{S1}$$

it may happen that the convergence of the GMRES algorithm is slow, i.e. the number of iterations required to ensure the convergence criterion is too large. This issue can be solved by transforming the linear system through the aid of a *preconditioner* $\mathcal{P}(\omega)$ that can be used to improve the convergence of the iterative procedure.[1] In our specific case, the preconditioner is defined as a matrix such that $\mathcal{P}(\omega) \approx (\mathbf{A} - z(\omega)\mathbf{I})$. The linear system in eq. S1 can be transformed by applying the preconditioner on the left and/or the right of the coefficient matrix. Thus, we can define a *left-preconditioned* linear system as:

$$[\mathcal{P}(\omega)]^{-1} (\mathbf{A} - z(\omega)\mathbf{I})\mathbf{q} = [\mathcal{P}(\omega)]^{-1} \mathbf{R}, \tag{S2}$$

or a *right-preconditioned* linear system as:

$$(\mathbf{A} - z(\omega)\mathbf{I}) [\mathcal{P}(\omega)]^{-1} \mathbf{z} = \mathbf{R}, \quad \mathbf{z} = \mathcal{P}(\omega)\mathbf{q}. \tag{S3}$$

As it can be easily noticed, the preconditioned linear systems are formally equivalent to the original linear system, but if the preconditioner $\mathcal{P}(\omega)$ is chosen wisely, the convergence rate of the GMRES algorithm can be strongly improved. However, it is worth remarking that the introduction of a preconditioner increases the computational cost per step of the iterative procedure because that the generation of the Krylov subspace basis vectors is affected and the convergence criterion is modified.[2] Therefore, a good preconditioner is such that the number of iterations to converge the modified linear system is reduced, but at the same time the application of $[\mathcal{P}(\omega)]^{-1}$ is cheap in order to reduce also the total computational cost, both in terms of time and storage requirement.

S1.1 Banded preconditioner

One of the possibilities to approximate the coefficient matrix **A** is to retain only the largest matrix elements, whose position clearly depends on the indexing of the atoms. The simplest approach is the *lexicographic* indexing, in which the FQ atoms are ordered according to their Cartesian coordinates, firstly with respect to the x coordinate, then y and lastly to the z coordinate (see Fig. S1a). Given this indexing, we have generated a logarithmic mesh of the matrix **A**, i.e. the matrix has been transformed through the expression

$$\mathcal{A}_{ij} = \log_{10} |A_{ij}| \,, \tag{S4}$$

The logarithmic mesh of the matrix **A** for the GD20 system described in Table 2 in the main text are reported in Fig. S1b.

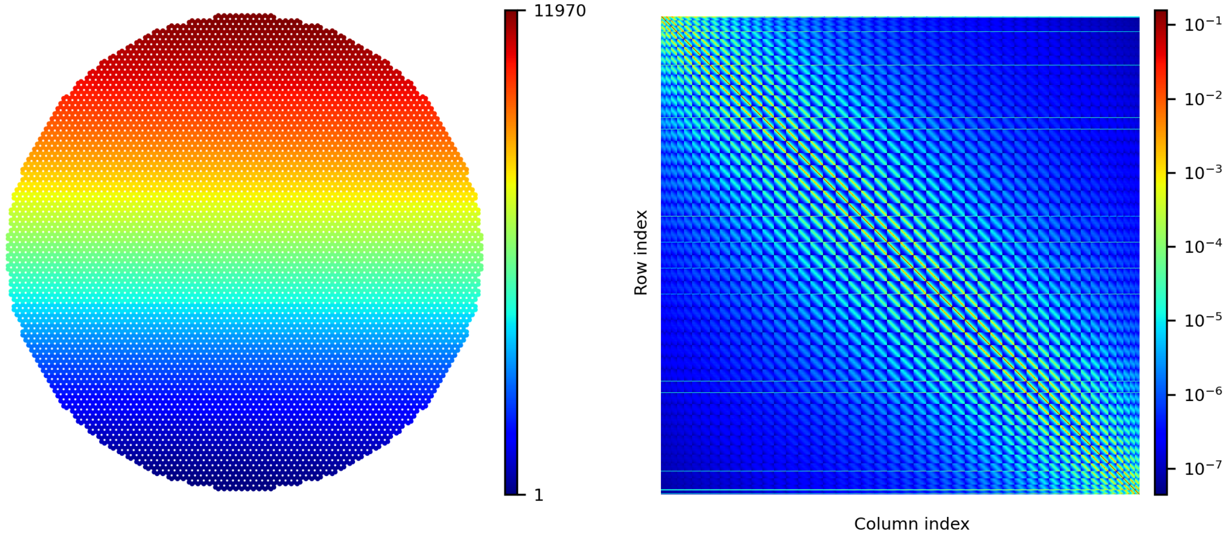

(a) Indexing (b) Logarithmic mesh

Figure S1: Graphical depiction of the lexicographic indexing of the GD system. In Fig. S1a the index of each FQ charge is represented. The mesh of the matrix $\mathcal{A}_{ij} = \log_{10} |A_{ij}|$ is reported in Fig. S1b.

As it can be noticed from Fig. S1b, the absolute values of matrix elements decay steadily with the distance from the diagonal, however, a sub-pattern due to the symmetry of the

system arises. Nevertheless the result obtained by using the logarithmic mesh suggests that a reasonable preconditioner can be built by retaining the diagonal and "a few" complete supradiagonal and subdiagonal elements of the matrix $\mathbf{A}$, and neglecting the other elements. By this, the most important matrix elements can be considered. For each iteration of the GMRES algorithm, a banded linear system needs to be solved. We can introduce the set of preconditioners

$$\mathcal{P}^{\mathrm{KU}}(\omega) = \mathbf{A}_{(\mathrm{KU})} - z(\omega)\mathbf{I}, \tag{S5}$$

where the matrix $\mathbf{A}_{(\mathrm{KU})}$ is defined as

$$\left(\mathbf{A}_{(\mathrm{KU})}\right)_{ij} = \begin{cases} A_{ij} & \text{if } \max(1, i-\mathrm{KU}) \leq j \leq \min(N, i+\mathrm{KU}) \\ 0 & \text{otherwise} \end{cases}, \tag{S6}$$

where N is the order of the matrix $\mathbf{A}$ and KU is a parameter determining the number of supradiagonals and subdiagonals†. Notice that with KU $= 0$ the preconditioner $\mathcal{P}^{0}(\omega)$ is a diagonal matrix.

The numerical performances of the band preconditioner (BP) have been tested on GD36 (see Table 2 in the main text). In particular, ωFQ response has been calculated for 200 frequencies (from 0.0 eV to 2.0 eV with a constant step of 0.01 eV), by imposing $\varepsilon = 1.51$ eV and $\tau = 170$ a.u. Both the left and right preconditioned linear system have been considered, with a convergence criterion of RMSE $< 10^{-5}$. The results are reported in Fig. S2.

It can be seen that if we consider KU ≥ 600 the number of iterations of the preconditioned linear system is less than the non preconditioned case. In particular, no significant differences in the performance of the algorithm between KU $= 600$ and KU $= 800$ or 1000 are reported. Therefore, we can conclude that for GD36 the right BP with KU $= 600$ is the

†The definition of the banded preconditioner through the KU parameter has been chosen according to the packed storage of banded matrices adopted in the LAPACK set of subroutines.[3] In general, a banded matrix is defined by the number of non-zero supradiagonals KU and the number of non-zero subdiagonals KL, but in our case we decided to put KU = KL therefore only the KU parameter is needed to define the matrix.

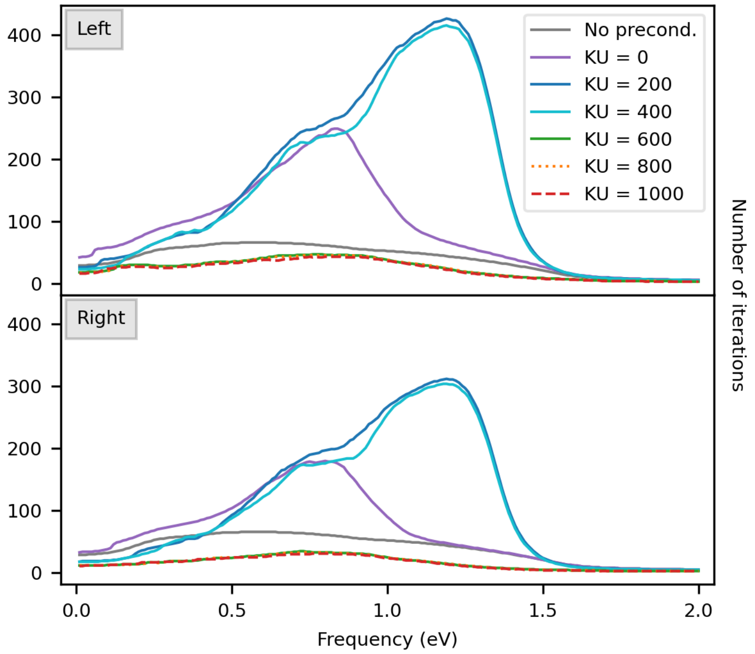

Figure S2: Number of iterations for the BAND preconditioner for the lexicographic-ordered GD36 system for different values of the KU parameter. The left preconditioning case is reported in the upper panel, while the right preconditioned case is reported in the bottom panel. The non preconditioned case (i.e. the standard ωFQ linear system) is the grey line.

best compromise between numerical quality and computational cost.

S1.1.1 Feasibility of the band preconditioning

As it has been shown in the main work, ωFQ can treat large-size nanostructures, with a number of atoms of about one million. By this, the numerical performances of the preconditioner have to be evaluated for systems of such dimensions. Thus, several issues need to be taken into account:

1. The preconditioning of the linear system is clearly more advantageous if less iterations are needed to achieve the convergence of the solution vector. The BP preconditioner performances strongly depend on the elements distribution of the matrix $\mathbf{A}$ (see Fig. S1b). Therefore, we have investigated another possible ordering of FQ atoms to emphasize the decaying property of the elements, i.e. such that the most important elements are concentrated closely to the main diagonal. In this way, the number of supradiag-

onals and subdiagonals needed to approximate the matrix **A** can be reduced. To this end, we have tested an approach based on "space-filling curves" which are curves able to map the whole two-dimensional unit square.[4] We chose the Hilbert space-filling curve, which is a mapping that preserves the locality fairly well.[5,6] The indexing of the FQ atoms of the GD20 system ordered through the Hilbert curve and the related logarithmic mesh of the matrix **A** have been depicted in Fig. S3a and Fig. S3b, respectively.

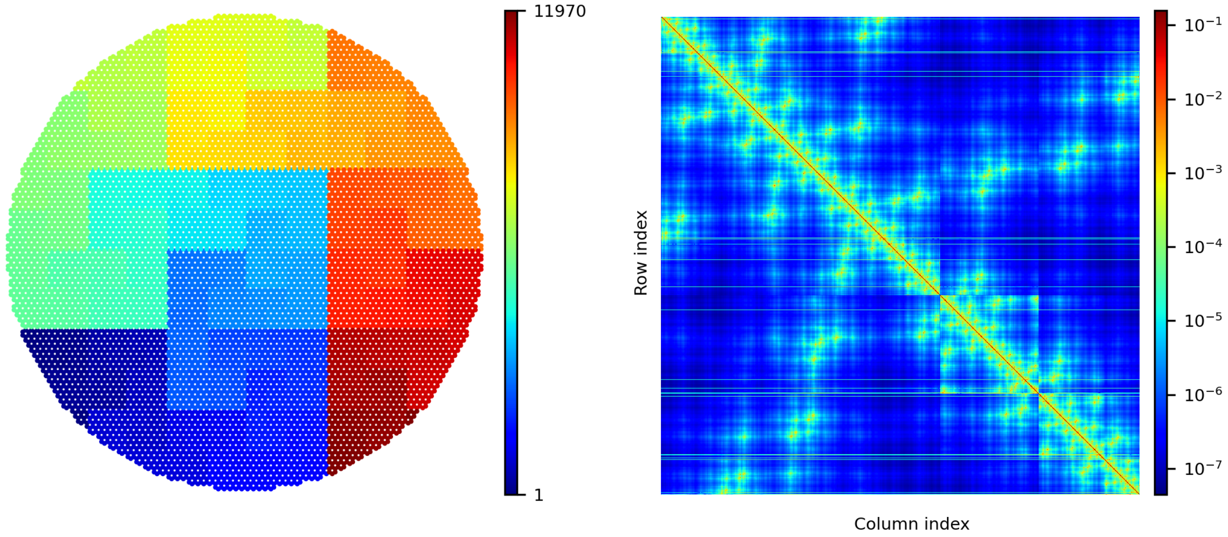

(a) Indexing

(b) Mesh

Figure S3: Graphical depiction of the Hilbert indexing of the GD system. In Fig. S3a the index of each FQ charge is represented. The mesh of the matrix $\mathcal{A}_{ij} = \log_{10}|A_{ij}|$ is reported in Fig. S3b.

From an inspection of Fig. S3b, we see that the density of non-negligible matrix elements close to the main diagonal is higher with respect to the lexicographic indexing (see Fig. S1b); as a trade-off, several accumulations of large matrix elements appeared at marginal positions of the **A** matrix with respect to the main diagonal.

In order to investigate the efficacy of the Hilbert-ordering as compared to the lexicographic indexing, we have tested the former ordering by applying ωFQ to the same system studied in Fig. S2. The results are reported in Fig. S4. It is worth remark-

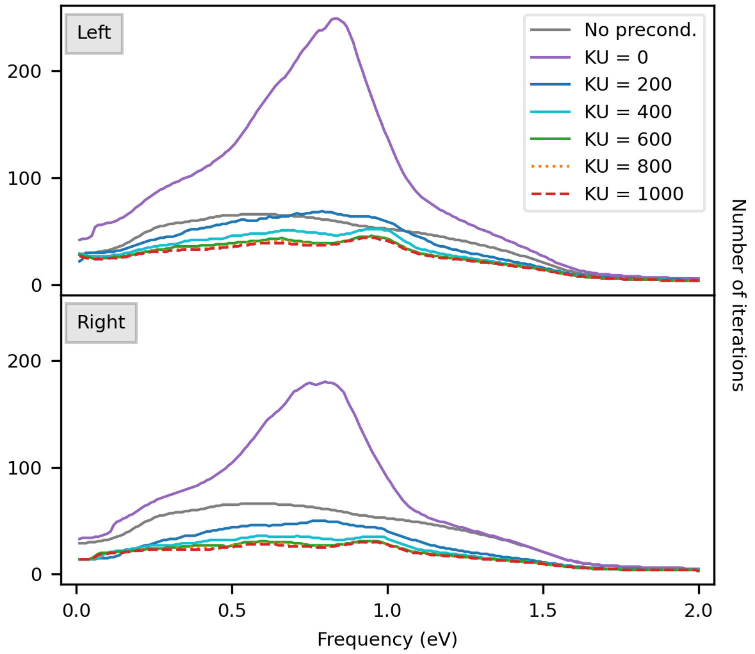

Figure S4: Number of iterations for the BP for the Hilbert-ordered GD36 system for different values of the KU parameter. The left preconditioning case is reported in the upper panel, while the right preconditioned case is reported in the bottom panel. The non preconditioned case (i.e. the standard ωFQ linear system) is the grey line.

ing that the number of iterations required to converge GMRES algorithm in the non preconditioned case is independent of the specific ordering of the atoms, i.e. the lexicographic and the Hilbert indexing do not affect the convergence rate of the iterative procedure. Moving to the preconditioned case, remarkably the Hilbert approach yields faster convergence of the preconditioner quality with the KU parameter. In fact, already with KU $= 400$ the number of iterations of the preconditioned linear system is less than the non preconditioned case for both the left and right cases. The average number of iterations required to achieve convergence over the 200 frequencies for GD36 ordered accordingly the lexicographic and Hilbert ordering with the KU $= 600$ BP is reported in Tab. S1.

Even if the Hilbert-ordering of the GD system induces a more favourable distribution of the matrix elements, the lexicographic-ordering is still the most advantageous with respect to the number of iterations with KU $= 600$.

Table S1: Average number of iterations to achieve convergence for the GMRES algorithm on the GD36 system with different orderings of the FQ atoms.

	Lexicographic	Hilbert
Non preconditioned	39	39
Left, KU = 600	24	26
Right, KU = 600	16	18

2. The preconditioner $\mathcal{P}^{\mathrm{KU}}(\omega)$ defined in eq. S5 explicitly depends on the external field frequency ω through the parameter $z(\omega)$. In order to calculate the preconditioner (and possibly a factorization of the latter) only once for all the investigated frequencies, a possible approach is to define a fixed-frequency preconditioner $\mathcal{P}^{\mathrm{KU}}(\omega')$ with $\omega' =$ constant. The fixed-frequency right BP has been tested on the same system studied above, by imposing KU = 600. In particular, three different values of ω' have been considered: 0.0 eV (the static case), 0.23 eV (the plasmon resonance frequency of the GD36 system) and 1.0 eV (the average frequency of the investigated range). The results are reported in Fig. S5.

   As it can be easily noticed, the fixed-frequency preconditioning is able to reduce the number of iterations as compared to the non preconditioned case, only for frequencies close to the value of ω', with a performance comparable to the frequency-dependent case. If the difference between ω' and the external field frequency ω is higher than 0.2 eV the number of iterations strongly increases, making the preconditioning counter-productive.

3. The application of $\left[\mathcal{P}^{\mathrm{KU}}(\omega)\right]^{-1}$ is the heaviest task of the preconditioning process. Since this involves the solution of a linear system, two approaches can be applied: direct methods (i.e. factorization of the preconditioner) or iterative techniques.

   - Direct methods: the preconditioner $\mathcal{P}^{\mathrm{KU}}$ has to be explicitly stored in memory and factorized through methods such as the LU decomposition. With KU = 600 and $N = 10^6$ the number of non-zero complex-valued elements of $\mathcal{P}(\omega)$ is about

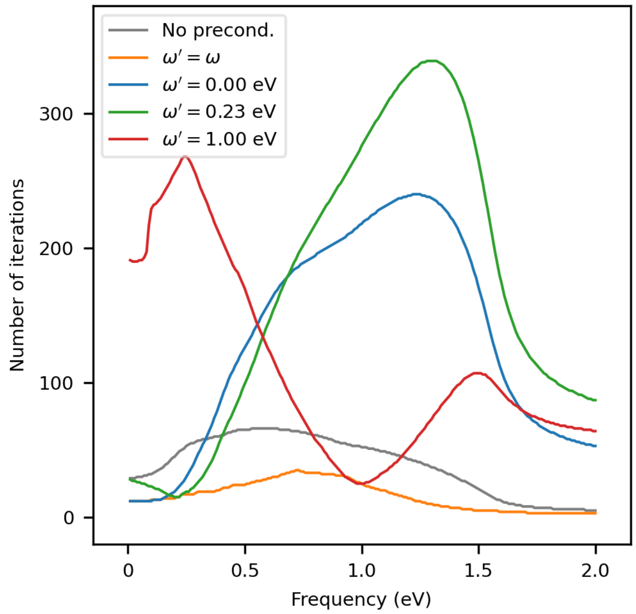

Figure S5: Number of iterations for the fixed-frequency right BP for the GD36 system, with KU = 600 and three different values of the fixed-frequency ω'. As a comparison, the non preconditioned case (grey line) and the frequency-dependent BP (orange line) have been reported.

$(2\text{KU} + 1)N = 1.2 \cdot 10^9$, which would require about 17 GB of RAM in packed storage. This memory demand could increase in the LU factorization due to the possible pivoting of the matrix to ensure the numerical stability. Moreover, the factorization of the $\mathcal{P}(\omega)$ has to be performed for each external field frequency ω since it has been shown that fixed-frequency BP is not able to reduce the number of iterations (see Fig. S5).

- Iterative methods: the linear system $\mathcal{P}(\omega)\mathbf{x} = \mathbf{y}$ can be solved in an approximate fashion by means of iterative techniques, in which the matrix-vector product can be calculated on-the-fly (see main text), and adopting a coarse convergence criterion. This means that the preconditioner at each step would be formally different: by this, the GMRES algorithm cannot be straightforwardly applied. This problem can be solved by adopting the "flexible GMRES" (FGMRES) method, which allows for a different preconditioning at each iterative step.[7] However, the storage

demand of FGMRES is higher, since also the preconditioned vectors have to be stored at each step. Finally, the efficiency of the preconditioner-related iterative solution has to be investigated in order to understand if the total computational time of the preconditioned linear system is less than the non preconditioned case.

4. All the above BP analysis has been performed on a 2D-system (GD36). However, as shown in the main text, ωFQ can be applied also to 3D nanostructured systems. Therefore, we have investigated the transferability of the performed analysis by applying the right BP with KU = 600 to two 3D systems, i.e. a sodium nanorod (NR) with a radius of 5 nm and a length of 20 nm and a sodium nanosphere (NS) with a radius of 45 Å, with a total number of atoms $N = 10444$ and $N = 10081$, respectively. Both systems have been ordered according to the lexicographic indexing. The calculations on the NR system have been performed with ωFQ parameters for sodium reported in a previous work,[8] for 150 frequencies in the range between 1.0 and 2.5 eV with a constant step of 0.01 eV and a RMSE threshold set to 10^{-5}. The number of iterations of the non preconditioned linear system and of the right-preconditioned linear system with $\mathcal{P}^{600}(\omega)$ are reported in Fig. S6.

   From an inspection of Fig. S6, it can be seen that in the lower part of the spectrum the right BP is able to slightly reduce the number of iterations. However, when ωFQ linear system becomes hard to converge with the GMRES algorithm, the preconditioner is not able to improve the convergence and the number of iterations increases similarly to the non preconditioned case.

   The calculations on the NS system have been performed with the same parameters as for the NR case. The ωFQ linear system has been solved for 200 frequencies in the range between 2.0 and 4.0 eV with a constant step of 0.01 eV and a RMSE threshold set to 10^{-5}. The number of iterations of the non preconditioned linear system and of the right-preconditioned linear system with $\mathcal{P}^{600}(\omega)$ have been reported in Fig.

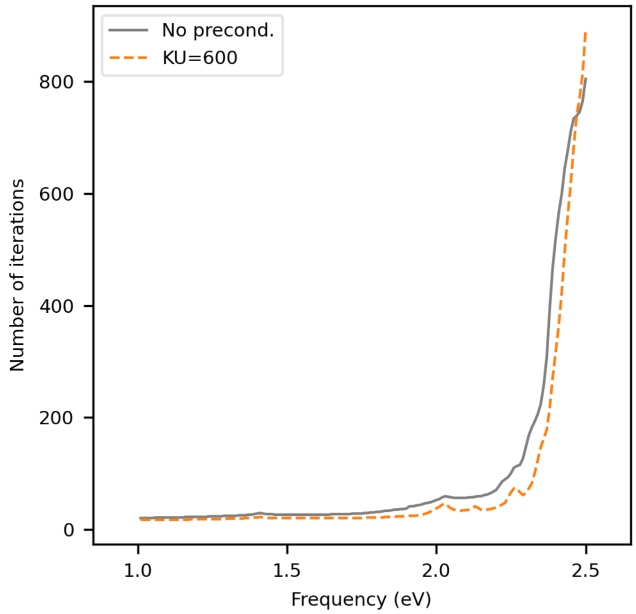

Figure S6: Number of iterations to converge the ωFQ linear system applied on the sodium NR system, for both the non preconditioned case (solid grey line) and with the right BP with KU = 600 (dashed orange line).

S7. From a comparison of Fig. S6 and Fig. S7 we see that a different topology of the nanostructured system induces a strongly different performance of the same preconditioner: in fact in the NS case with the increase of the external field frequency the preconditioned linear system is harder to converge, and after a frequency of about 3.6 eV the convergence is not achieved in 1000 iterations. At the same time the non preconditioned case shows an almost uniform convergence in the highest part of the spectrum.

As a final remark, the shape of the matrix $\mathbf{A}$ strongly depends on the spatial distribution of the atoms of the plasmonic substrate, therefore we cannot assume BP to be a good preconditioner even for more complicated and less regular plasmonic substrates, and the optimal value of the KU parameter needs to be specified for each nanostructure.

By taking a look to all these issues, it can be stated that the BP is not able to give a

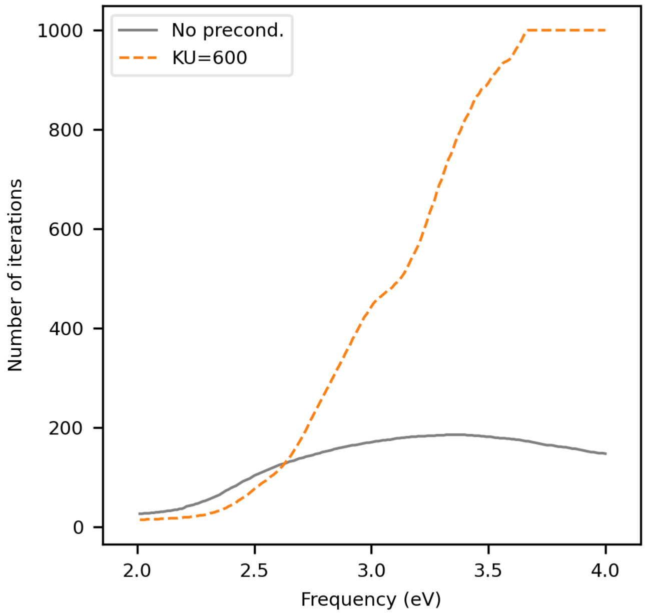

Figure S7: Number of iterations to converge the ωFQ linear system applied on the sodium NS system, for both the non preconditioned case (solid grey line) and with the right BP with $\mathrm{KU} = 600$ (dashed orange line).

computational boost to the GMRES algorithm applied to the ωFQ linear system.

S1.2 Gauss-Seidel preconditioner

The second tested preconditioner is the symmetric Gauss-Seidel preconditioner (GS), which belongs to the class of Symmetric Successive Over-Relaxation (SSOR) preconditioners.[9–11] In particular, it is defined as

$$\mathcal{P}(\omega) = (\mathbf{L} + \tilde{\mathbf{D}}(\omega)) \left[\tilde{\mathbf{D}}(\omega)\right]^{-1} (\tilde{\mathbf{D}}(\omega) + \mathbf{U}), \tag{S7}$$

where $\mathbf{L}$ and $\mathbf{U}$ are the strictly lower triangular and strictly upper triangular part of the matrix $\mathbf{A}$, while $\tilde{\mathbf{D}}$ is the diagonal of the total coefficient matrix of the ωFQ linear system, i.e.

$$\tilde{D}_{ij}(\omega) = \left[A_{ii} - z(\omega)\right] \delta_{ij} \tag{S8}$$

The numerical performances of the GS preconditioner have been tested with 83 frequencies from 0.0 eV to 0.82 eV with a constant step of 0.01 eV, on the GD36 system with the same parameters exploited above. The results are reported in Fig. S8.

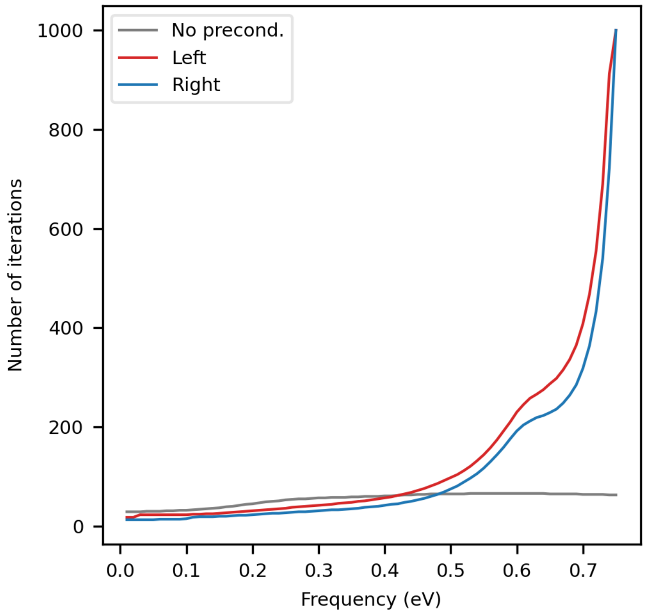

Figure S8: Number of iterations for the preconditioned ωFQ linear system of the GD36 system through the GS preconditioner, for both the left (red line) and right (blue line) case.

As it can be noticed, in the lowest part of the frequency range the GS preconditioner slightly reduces of the number of iterations, while over 0.45 eV the non preconditioned linear system converges faster. As noticed before for BP, the right preconditioning is slightly more efficient than the left one. However, above $\omega = 0.75$ eV the preconditioned linear system fails to converge in 1000 iterations.

S1.3 Nearest-Neighbours preconditioner

We finally tested a Nearest-Neighbours preconditioner (NN), which has been introduced by assuming that the total electric field experienced by the i-th FQ can be approximated by retaining the interactions with the closest atoms only, as defined by means of a threshold parameter δ. Given the definition of the matrix $\mathbf{A}$ reported in Equation 17 in the main text,

i.e.:

$$\mathbf{A} = (\overline{\mathbf{K}}^{\text{tot}} - \mathbf{P})\mathbf{D}. \tag{S9}$$

we can introduce the following submatrices:

$$D_{ij}^{\delta} = \begin{cases} D_{ij} & \text{if } |\mathbf{r}_i - \mathbf{r}_j| \leq \delta, \\ 0 & \text{otherwise} \end{cases} \tag{S10}$$

$$\left[K_{ij}^{\text{tot}}\right]^{\delta} = \begin{cases} K_{ij}^{\text{tot}} & \text{if } |\mathbf{r}_i - \mathbf{r}_j| \leq \delta, \\ 0 & \text{otherwise} \end{cases} \tag{S11}$$

where $\mathbf{r}_i$ is the position of the i-th atom. By this, we can define the NN preconditioner as

$$\mathcal{P}^{\delta}(\omega) = (\left[\mathbf{K}^{\text{tot}}\right]^{\delta} - \mathbf{P})\mathbf{D}^{\delta} - z(\omega)\mathbf{I}. \tag{S12}$$

The distance parameter δ is chosen by taking into account the carbon-carbon bond length in graphene ($d = 0.142$ nm).[12] In particular, the values $\delta = d$, $\delta = 2d$ and $\delta = 3d$ have been considered, and the related preconditioners have been labeled as NN1, NN2 and NN3, respectively.

The numerical performances of NN1, NN2 and NN3 have been tested on the GD36 system with the same parameters exploited above. The results are not shown, since GMRES fails to converge in 1000 iterations independently of the frequency ω and of the parameter δ. A possible explanation of the lack of convergence is that ωFQ is intrinsically able to treat non-local effects.[8]

S2 Results

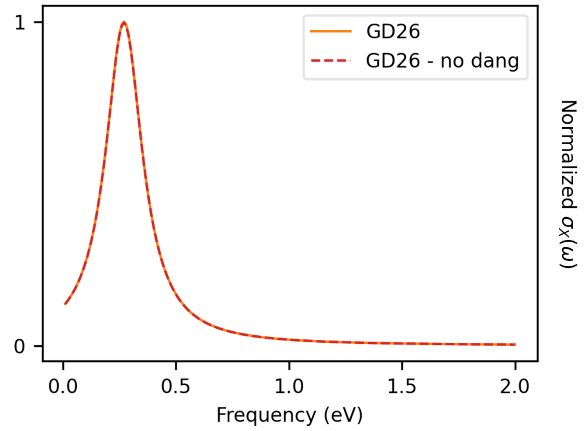

Figure S9: Longitudinal absorption cross section of a graphene disk with diameter equal to 26 nm (GD26) as calculated by including or discarding the dangling bonds.

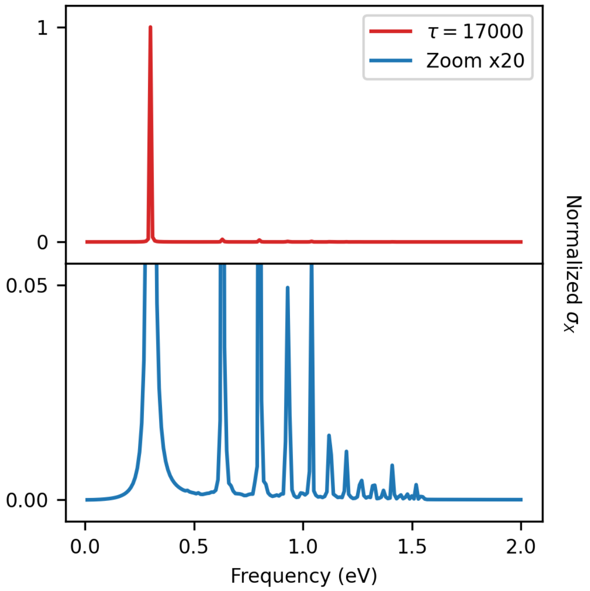

Figure S10: Longitudinal absorption cross section of a graphene disk with diameter equal to 20 nm (GD20) as calculated by imposing $\tau = 17000$ a.u.

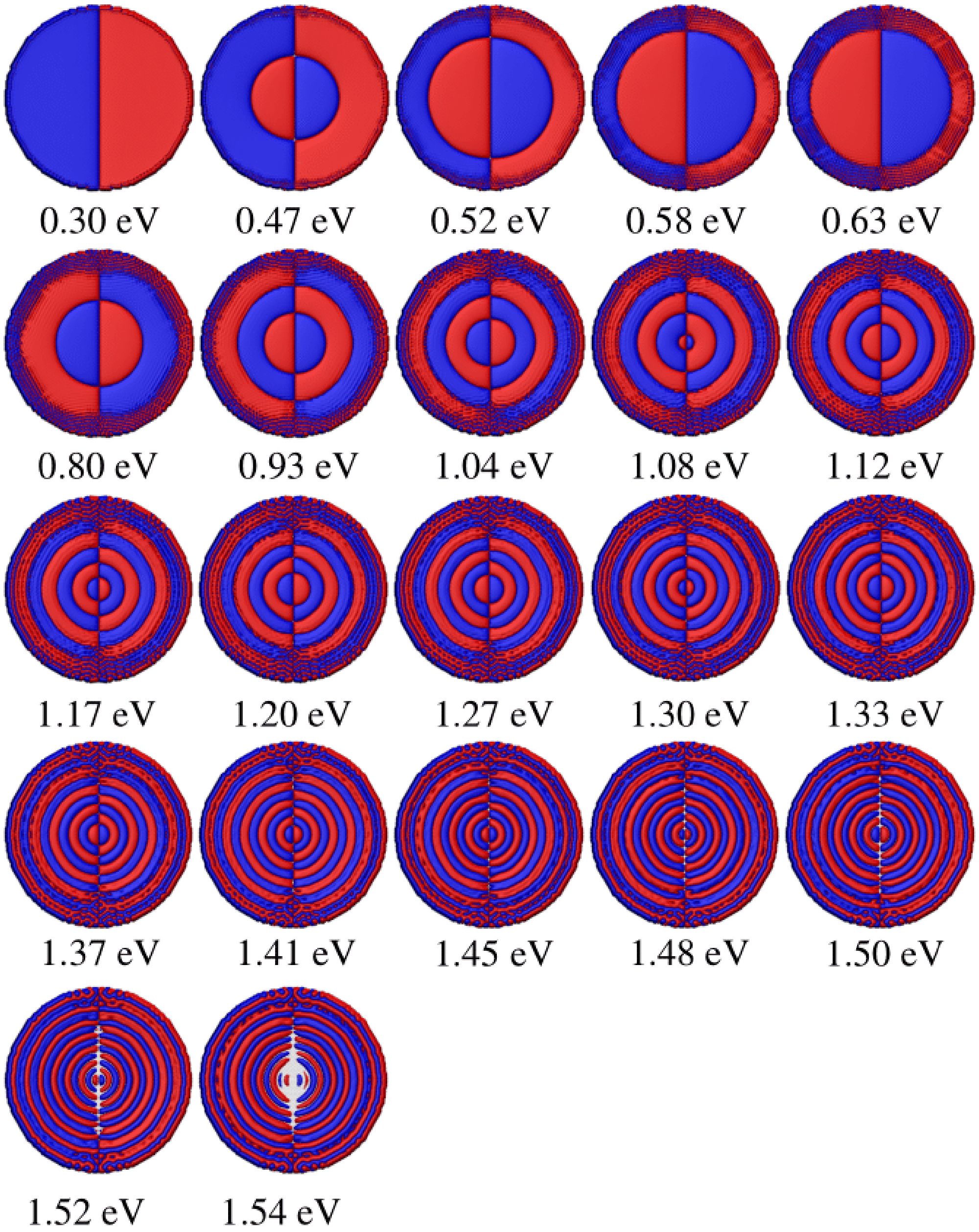

Figure S11: Graphical depiction of GD20 plasmon densities calculated at PRFs highlighted in Fig. 2 in the main text. The isovalue is set to 2e-7.

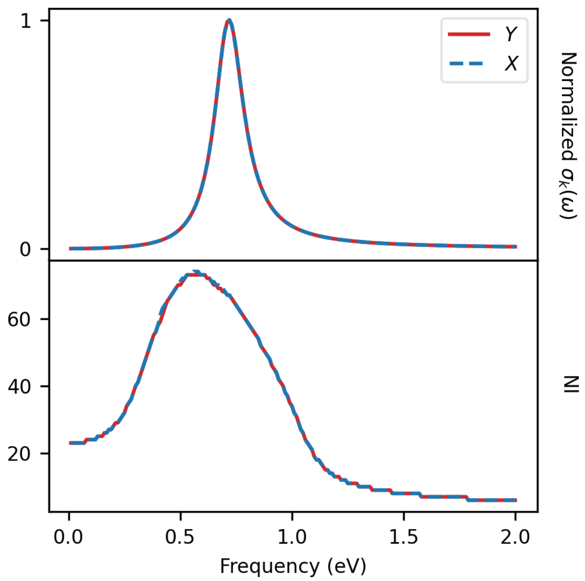

Figure S12: CNT50 σ_X and σ_Y and the associated number of iterations.

Table S2: Plasmon resonance frequencies (PRFs) for the systems reported in the main text.

	PRF X (eV)	PRF Z (eV)
CNT50	0.72	0.08
CNT100	0.72	0.05
CNT200	0.72	0.04
CNT300	0.72	0.03
CNT1	0.72	0.08
CNT2	0.53	0.09
CNT3	0.43	0.10
CNT4	0.37	0.11
GD20	0.30	-
GD26	0.27	-
GD32	0.24	-
GD36	0.23	-

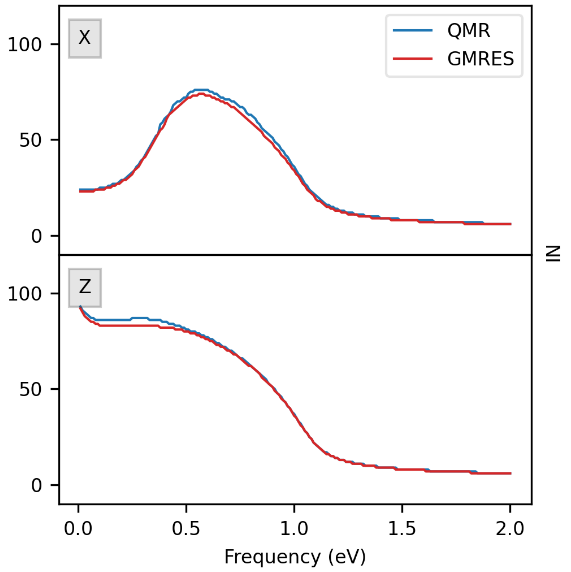

Figure S13: GMRES and QMR CNT300 convergence rate. Both longitudinal (bottom) and transverse (top) polarizations of the external field are reported.

S3 Upper bound

Let us consider the ωFQ linear system, i.e.:

$$(\mathbf{A} - z(\omega)\mathbf{I})\mathbf{q}^k = \mathbf{R}^k, \tag{S13}$$

where ω is the external field frequency polarized along the k direction. Once we have solved the linear system, we can calculate the longitudinal absorption cross section along the k axis as:

$$\sigma^k(\omega) = \sum_i^N \frac{k_i}{E^0} \cdot \mathrm{Im}(q_i^k), \tag{S14}$$

where k_i is the coordinate of the i-th charge along the k axis, E^0 is the intensity of the external field and $\mathrm{Im}(q_i^k)$ is the imaginary part of the i-th charge. The isotropic absorption cross section is simply the average of the three longitudinal absorption cross sections, that is:

$$\sigma(\omega) = \frac{1}{3} \sum_{k=x,y,z} \sigma^k(\omega). \tag{S15}$$

Solving eq. S13, the numerical error can be estimated by the condition number κ of the matrix $(A - z(\omega)I)$ through the expression:

$$\frac{\left\|\delta\mathbf{q}^k\right\|}{\left\|\mathbf{q}^k\right\|} \leq \kappa \frac{\left\|\delta\mathbf{R}^k\right\|}{\left\|\mathbf{R}^k\right\|}, \tag{S16}$$

where $\delta\mathbf{q}^k$ is the error of the solution vector and $\delta\mathbf{R}^k$ is the residual vector, i.e.:

$$\delta\mathbf{R}^k = \mathbf{R}^k - (\mathbf{A} - z\mathbf{I})(\mathbf{q}^k + \delta\mathbf{q}^k).$$

In other words κ is the maximum ratio between the relative errors of the solution and the right-hand side vectors, both calculated with the same norm. We will use the 1-norm, defined as:

$$\left\|\mathbf{q}^k\right\| = \sum_i^N \left|q_i^k\right|. \tag{S17}$$

The absolute error is defined as the absolute difference between the calculated value $\mathbf{q}$ and the exact value $\overline{\mathbf{q}}$, i.e.:

$$\left\|\delta\mathbf{q}^k\right\| = \sum_i^N \left|\delta q_i^k\right| = \sum_i^N \left|q_i^k - \overline{q_i^k}\right|. \tag{S18}$$

In order to calculate a reliable upper bound for the error on the isotropic absorption cross section, from eq. S16 we can estimate the absolute error on the solution vector as:

$$\left\|\delta\mathbf{q}^k\right\| \leq \kappa \left\|\delta\mathbf{R}^k\right\| \cdot \frac{\left\|\mathbf{q}^k\right\|}{\left\|\mathbf{R}^k\right\|}. \tag{S19}$$

The absolute error on the longitudinal absorption cross section can be calculated as:

$$\begin{aligned} \delta\sigma^k = \left|\sigma^k - \overline{\sigma^k}\right| = \left|\sum_i^N \frac{k_i}{E^0}\mathrm{Im}(q_i^k - \overline{q_i^k})\right| \leq \\ \leq \sum_i^N \frac{k_i}{E^0}\left|\mathrm{Im}(q_i^k - \overline{q_i^k})\right| \leq \sum_i^N \frac{k_i}{E^0}\left|\delta q_i^k\right| \end{aligned} \tag{S20}$$

where we have assumed $k_i \geq 0$ for all charges, and exploited the triangle inequality property of the vector norm. Thus, an upper bound on the relative error on the isotropic absorption cross section can be written as:

$$\delta\sigma \leq \frac{1}{3E^0\sigma} \sum_{k=x,y,z} \sum_{i}^{N} k_i \left|\delta q_i^k\right|, \tag{S21}$$

under the constraint that

$$\sum_{i}^{N} \left|\delta q_i^k\right| \leq \kappa \frac{\left\|\mathbf{q}^k\right\|}{\left\|\mathbf{R}^k\right\|} \cdot \left\|\delta \mathbf{R}^k\right\|. \tag{S22}$$

At this point we can introduce two approximations:

1. we assume that absolute errors on the solution charges are evenly distributed, i.e.

$$\left|\delta q_i^k\right| = \frac{1}{N} \left\|\delta \mathbf{q}^k\right\|; \tag{S23}$$

2. each residual vector element is equal to the machine error (within the double precision floating point format $\varepsilon \approx 10^{-16}$). By this, the norm of the residual vector is:

$$\left\|\delta \mathbf{R}^k\right\| = \sum_{i}^{N} \left|\delta R_i^k\right| \approx \sum_{i}^{N} \varepsilon = N\varepsilon. \tag{S24}$$

Under these assumptions, the relative error on the isotropic absorption cross section becomes:

$$\delta\sigma \lessapprox \frac{\kappa N \varepsilon}{3E^0\sigma} \sum_{k=x,y,z} k_N \frac{\left\|\mathbf{q}^k\right\|}{\left\|\mathbf{R}^k\right\|}, \tag{S25}$$

where k_N is the average position along the k axis of the ωFQ charges.

S4 Computational time

The direct solution has been computed through an LU factorization with partial pivoting and row interchanges of the $\mathbf{A} - z\mathbf{I}$ matrix introduced in eq. S13, by means of the ZGETRF subroutine provided by the LAPACK software package.[13]

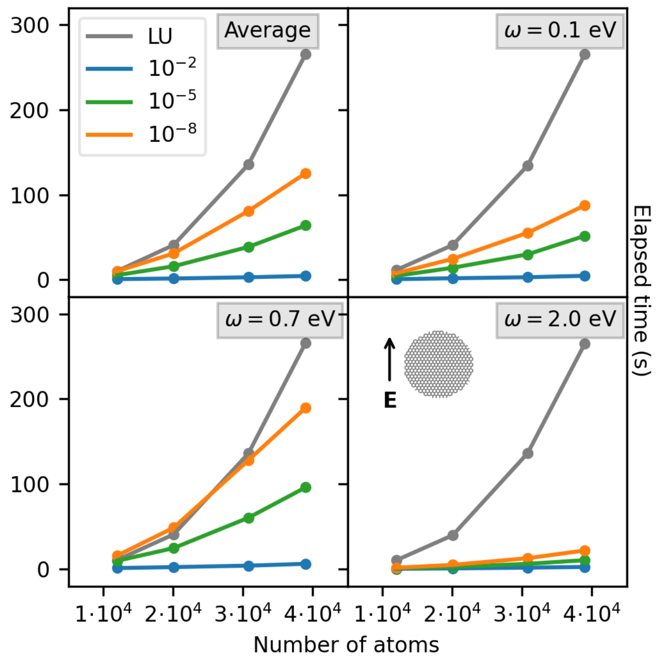

Figure S14: Computational time required to solve the ωFQ linear system as a function of the number of atoms in GD structures with the LU-based algorithm and QMR. Average (top, left), $\omega = 0.1$ eV (top, right), $\omega = 0.7$ eV (bottom, left) and $\omega = 2.0$ eV (bottom, right).

If we set RMSE $= 10^{-12}$, the QMR algorithm fails to converge for each structure and frequency in the chosen range, because of a breakdown of the no-look-ahead Lanczos procedure adopted in the algorithm proposed by Freund and Nachtigal.[14] Such breakdowns can be avoided by resorting to look-ahead techniques, but they have not been considered in this work.

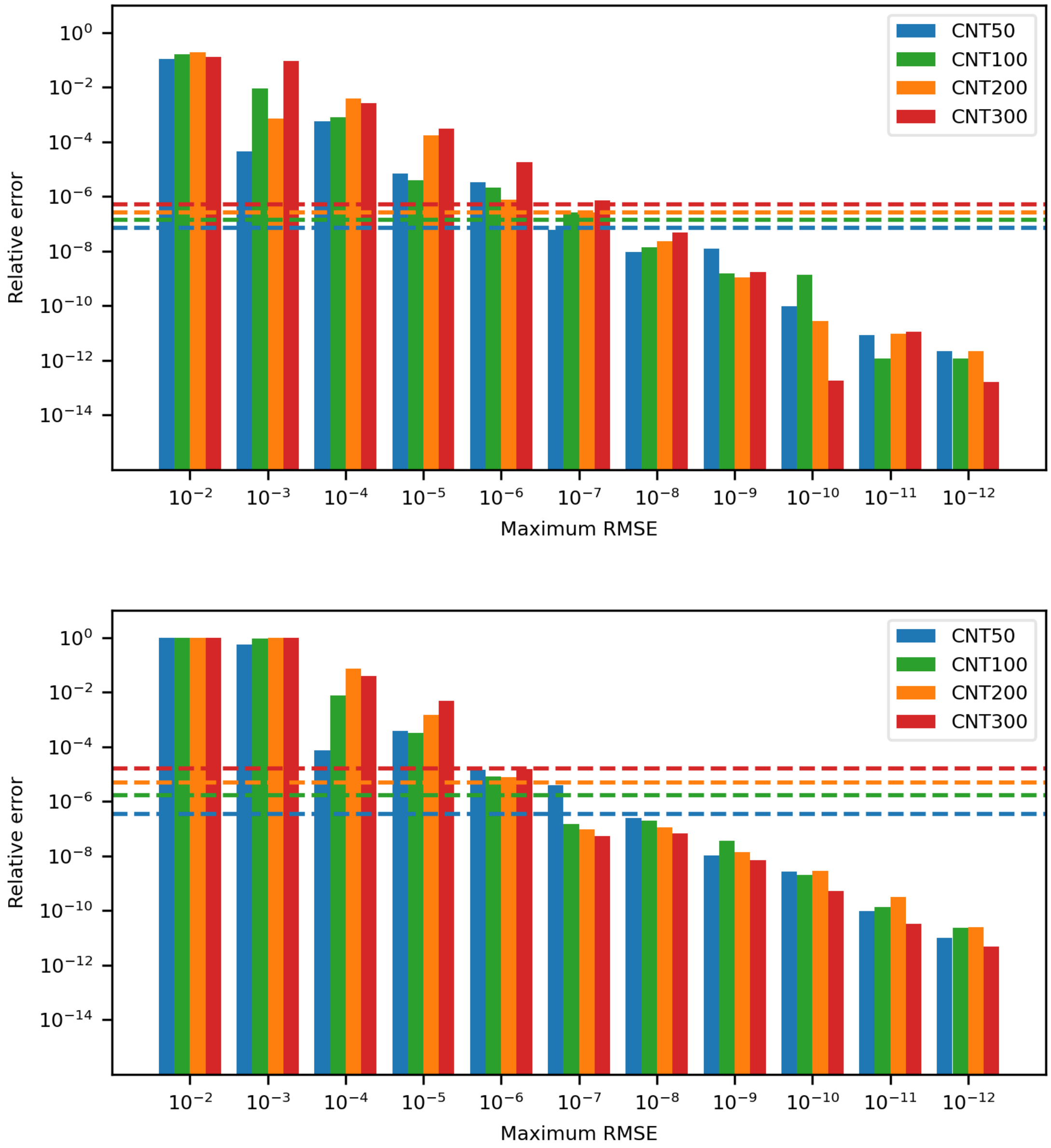

Figure S15: Average relative errors for CNT50, CNT100, CNT200 and CNT300 (see Table 1 in the main text) of the GMRES iterative solution with respect to LU factorization of the coefficient matrix at different choices of the RMSE. Dashed lines indicate an approximated upper bound of the intrinsic precision associated with the inversion algorithm. Both longitudinal (bottom) and transverse (top) polarizations are considered.

Table S3: GD20 computational timings (seconds) as obtained by using LU or GMRES algorithms. Different RMSE values are considered.

Frequency (eV)	LU	GMRES			
		10^{-2}	10^{-5}	10^{-8}	10^{-12}
0.1	11.591	0.387	2.728	5.593	11.165
0.2	10.407	0.505	4.233	8.611	19.750
0.3	10.336	0.549	6.485	13.693	24.710
0.4	9.981	0.525	6.016	15.428	29.530
0.5	10.006	0.510	6.427	15.377	28.278
0.6	10.545	0.547	7.186	14.490	29.349
0.7	10.573	0.499	7.272	14.600	29.991
0.8	9.928	0.526	6.928	15.123	29.289
0.9	10.168	0.448	5.929	12.567	26.227
1.0	10.121	0.464	4.469	10.665	24.886
1.1	10.369	0.392	4.410	10.050	22.778
1.2	10.256	0.318	3.972	8.605	18.470
1.3	9.899	0.244	3.657	7.531	15.114
1.4	10.139	0.258	2.853	5.725	10.854
1.5	10.171	0.258	1.684	3.690	7.219
1.6	11.102	0.284	0.957	1.961	3.582
1.7	9.753	0.275	0.684	1.258	2.137
1.8	10.174	0.290	0.605	1.066	1.530
1.9	10.698	0.280	0.620	0.983	1.450
2.0	10.298	0.279	0.575	0.899	1.287

Table S4: GD26 computational timings (seconds) as obtained by using LU or GMRES algorithms. Different RMSE values are considered.

Frequency (eV)	LU	GMRES			
		10^{-2}	10^{-5}	10^{-8}	10^{-12}
0.1	40.959	0.978	8.155	18.424	36.078
0.2	41.041	1.000	12.456	28.941	63.451
0.3	41.647	1.366	17.384	42.597	94.115
0.4	41.057	1.332	18.325	46.981	104.533
0.5	39.926	1.374	19.453	49.349	110.049
0.6	41.010	1.175	19.937	50.315	110.103
0.8	41.616	0.978	18.312	44.582	100.563
0.9	40.886	0.976	16.373	40.993	92.888
1.0	41.390	0.784	14.992	36.778	84.886
1.1	40.820	0.785	13.218	33.510	75.758
1.2	41.297	0.596	11.582	28.223	64.398
1.3	40.818	0.630	9.601	23.297	51.410
1.4	40.759	0.633	7.218	17.166	37.818
1.5	40.693	0.630	4.784	10.741	23.152
1.6	41.557	0.627	2.682	5.444	11.548
1.7	41.119	0.605	1.868	3.557	7.030
1.8	41.051	0.625	1.722	2.813	5.306
1.9	40.374	0.588	1.507	2.425	4.523
2.0	39.776	0.623	1.351	2.220	3.936

Table S5: GD32 computational timings (seconds) as obtained by using LU or GMRES algorithms. Different RMSE values are considered.

Frequency (eV)	LU	GMRES			
		10^{-2}	10^{-5}	10^{-8}	10^{-12}
0.1	134.460	1.692	16.432	32.687	62.734
0.2	135.312	1.759	23.883	49.980	102.017
0.3	135.425	2.442	30.804	68.966	143.004
0.4	134.959	2.974	33.769	74.229	157.925
0.5	135.690	2.326	35.722	80.763	166.879
0.6	135.437	1.963	36.063	80.684	169.631
0.7	136.059	2.043	35.290	78.754	170.929
0.8	135.471	2.069	32.750	75.101	163.397
0.9	135.970	1.592	29.296	69.241	152.897
1.0	135.677	1.640	27.383	64.123	142.028
1.1	136.038	1.206	24.521	58.371	130.741
1.2	135.421	1.301	21.669	51.624	116.145
1.3	136.133	1.173	18.526	44.221	95.230
1.4	136.745	1.214	14.364	33.549	74.069
1.5	135.814	1.335	9.606	23.058	47.466
1.6	135.100	1.378	5.764	12.467	24.900
1.7	137.483	1.319	4.001	8.630	15.887
1.8	136.263	1.272	3.445	6.608	12.590
1.9	135.556	1.247	3.140	5.943	10.376
2.0	136.201	1.208	2.684	5.087	9.490

Table S6: GD36 computational timings (seconds) as obtained by using LU or GMRES algorithms. Different RMSE values are considered.

Frequency (eV)	LU	GMRES			
		10^{-2}	10^{-5}	10^{-8}	10^{-12}
0.1	265.195	2.622	22.325	47.982	85.989
0.2	265.153	2.573	33.340	70.135	133.502
0.3	265.514	2.873	43.382	93.038	182.672
0.4	264.550	3.832	45.919	103.581	204.752
0.5	264.877	4.007	50.254	109.347	218.667
0.6	264.898	3.024	50.636	111.661	221.609
0.7	265.968	3.420	49.407	109.452	226.117
0.8	265.722	2.589	45.729	103.523	220.279
0.9	266.025	2.498	42.411	96.456	210.180
1.0	266.218	2.644	39.340	88.523	196.798
1.1	264.851	2.108	37.478	82.604	181.551
1.2	265.049	2.033	31.950	71.666	162.342
1.3	266.167	1.942	27.931	60.930	136.317
1.4	265.714	2.112	22.597	48.521	107.567
1.5	263.865	2.016	16.009	33.506	74.019
1.6	265.547	1.909	9.735	19.417	40.273
1.7	266.859	2.024	6.577	11.917	25.395
1.8	266.165	2.048	5.878	10.204	21.025
1.9	265.869	2.136	5.438	9.018	17.457
2.0	264.948	1.933	4.756	7.976	15.704

Table S7: GD20 computational timings (seconds) as obtained by QMR algorithm. Different RMSE values are considered.

Frequency (eV)	QMR		
	10^{-2}	10^{-5}	10^{-8}
0.1	0.764	4.713	7.797
0.2	1.089	6.421	10.599
0.3	2.395	8.195	16.370
0.4	2.095	8.135	16.962
0.5	1.961	8.989	16.716
0.6	2.008	9.592	15.692
0.7	1.089	9.652	15.784
0.8	0.953	8.306	16.859
0.9	0.720	7.605	14.650
1.0	0.714	7.285	12.461
1.1	0.632	6.821	12.420
1.2	0.416	6.183	11.302
1.3	0.252	5.266	9.968
1.4	0.233	4.346	8.569
1.5	0.274	3.058	5.872
1.6	0.275	1.904	3.431
1.7	0.258	1.323	2.332
1.8	0.232	0.991	1.864
1.9	0.235	0.851	1.605
2.0	0.249	0.904	1.458

Table S8: GD26 computational timings (seconds) as obtained by QMR algorithm. Different RMSE values are considered.

Frequency (eV)	QMR		
	10^{-2}	10^{-5}	10^{-8}
0.1	1.867	14.208	24.679
0.2	2.394	18.590	33.918
0.3	3.421	23.507	46.139
0.4	2.999	24.412	47.190
0.5	2.877	24.845	48.433
0.6	2.608	24.796	48.593
0.7	2.278	24.599	48.567
0.8	1.944	23.788	47.077
0.9	1.803	22.216	44.742
1.0	1.297	21.830	43.085
1.1	1.243	20.498	40.512
1.2	0.736	18.410	36.952
1.3	0.728	16.569	31.834
1.4	0.751	13.467	26.279
1.5	0.690	9.472	18.341
1.6	0.762	5.536	11.325
1.7	0.704	3.948	7.827
1.8	0.725	3.071	6.121
1.9	0.699	2.796	5.142
2.0	0.710	2.306	4.871

Table S9: GD32 computational timings (seconds) as obtained by QMR algorithm. Different RMSE values are considered.

Frequency (eV)	QMR		
	10^{-2}	10^{-5}	10^{-8}
0.1	3.086	30.002	55.356
0.2	4.065	43.149	75.457
0.3	6.562	52.686	99.874
0.4	6.758	55.114	106.292
0.5	5.138	58.214	117.440
0.6	5.285	59.265	122.017
0.7	3.936	60.346	128.033
0.8	4.126	58.629	129.862
0.9	2.890	55.762	124.609
1.0	2.914	54.601	117.801
1.1	1.604	52.359	115.906
1.2	1.557	46.775	104.815
1.3	1.648	41.672	92.347
1.4	1.571	35.702	77.327
1.5	1.731	24.753	54.285
1.6	1.635	15.720	31.344
1.7	1.635	9.020	21.740
1.8	1.552	8.276	17.029
1.9	1.495	7.118	15.443
2.0	1.628	6.117	12.715

Table S10: GD36 computational timings (seconds) as obtained by QMR algorithm. Different RMSE values are considered.

Frequency (eV)	QMR		
	10^{-2}	10^{-5}	10^{-8}
0.1	4.722	51.707	87.568
0.2	4.821	67.841	120.102
0.3	10.816	83.531	155.461
0.4	9.006	90.604	168.085
0.5	8.491	97.726	178.767
0.6	6.582	97.418	185.332
0.7	6.138	95.978	189.647
0.8	4.547	94.762	189.570
0.9	4.910	91.752	186.619
1.0	4.304	90.695	181.975
1.1	2.689	85.175	176.355
1.2	2.484	79.007	164.797
1.3	2.897	74.275	149.388
1.4	2.465	59.919	123.048
1.5	2.574	41.843	88.372
1.6	2.619	25.346	51.609
1.7	2.970	15.598	33.053
1.8	2.795	14.658	31.117
1.9	2.768	11.889	25.086
2.0	2.470	10.375	21.785

Table S11: Geometrical parameters of CNT1M, GD1M and NR1M (see main text).

ID	Length (nm)	Diameter (nm)	Number of atoms
CNT1M	1500	5.46	983744
GD1M	-	182	996006
NR1M	90	20	1009102

References

(1) Chen, K. *Matrix preconditioning techniques and applications*; Cambridge University Press, 2005.

(2) Frayssé, V.; Giraud, L.; Gratton, S.; Langou, J. *A set of GMRES routines for real and complex arithmetics on high performance computers: Technical Report TR*; 2003; public domain software available on www.cerfacs/algor/Softs.

(3) Anderson, E.; Bai, Z.; Bischof, C.; Blackford, L. S.; Demmel, J.; Dongarra, J.; Du Croz, J.; Greenbaum, A.; Hammarling, S.; McKenney, A., et al. *LAPACK Users' guide*; SIAM, 1999.

(4) Sagan, H. *Space-filling curves*; Springer Science & Business Media, 2012.

(5) Hilbert, D. *Dritter Band: Analysis· Grundlagen der Mathematik· Physik Verschiedenes*; Springer, 1935; pp 1–2.

(6) Moon, B.; Jagadish, H. V.; Faloutsos, C.; Saltz, J. H. Analysis of the clustering properties of the hilbert space-filling curve. *IEEE Transactions on knowledge and data engineering* **2001**, *13*, 124–141.

(7) Saad, Y. A flexible inner-outer preconditioned GMRES algorithm. *SIAM Journal on Scientific Computing* **1993**, *14*, 461–469.

(8) Giovannini, T.; Rosa, M.; Corni, S.; Cappelli, C. A classical picture of subnanometer junctions: an atomistic Drude approach to nanoplasmonics. *Nanoscale* **2019**, *11*, 6004–6015.

(9) Axelsson, O. A class of iterative methods for finite element equations. *Computer Methods in Applied Mechanics and Engineering* **1976**, *9*, 123–137.

(10) Axelsson, O. A survey of preconditioned iterative methods for linear systems of algebraic equations. *BIT Numerical Mathematics* **1985**, *25*, 165–187.

(11) Axelsson, O.; Barker, V. A. *Finite element solution of boundary value problems: theory and computation*; SIAM, 2001.

(12) Cooper, D. R.; D'Anjou, B.; Ghattamaneni, N.; Harack, B.; Hilke, M.; Horth, A.; Majlis, N.; Massicotte, M.; Vandsburger, L.; Whiteway, E., et al. Experimental review of graphene. *International Scholarly Research Notices* **2012**, *2012*.

(13) Wang, E.; Zhang, Q.; Shen, B.; Zhang, G.; Lu, X.; Wu, Q.; Wang, Y. *High-Performance Computing on the Intel® Xeon Phi™* ; Springer, 2014; pp 167–188.

(14) Freund, R. W.; Nachtigal, N. M. Software for simplified Lanczos and QMR algorithms. *Appl. Numer. Math.* **1995**, *19*, 319–341.
